# Behavioural phenotypes of intrinsic motivation in schizophrenia determined by cluster analysis of objectively quantified real-world performance

**DOI:** 10.1038/s41537-022-00294-0

**Published:** 2022-10-21

**Authors:** Ishraq Siddiqui, Gary Remington, Sarah Saperia, Susana Da Silva, Paul J. Fletcher, Aristotle N. Voineskos, Konstantine K. Zakzanis, George Foussias

**Affiliations:** 1 Schizophrenia Division and Campbell Family Mental Health Research Institute, Centre for Addiction and Mental Health, Toronto, ON Canada; 2 Institute of Medical Science, Faculty of Medicine, University of Toronto, Toronto, ON Canada; 3 Slaight Family Centre for Youth in Transition, Centre for Addiction and Mental Health, Toronto, ON Canada; 4 Department of Psychiatry, University of Toronto, Toronto, ON Canada; 5 Department of Psychology, University of Toronto Scarborough, Toronto, ON Canada; 6 Preclinical Research and Campbell Family Mental Health Research Institute, Centre for Addiction and Mental Health, Toronto, ON Canada; 7 Department of Psychology, University of Toronto, Toronto, ON Canada

**Keywords:** Schizophrenia, Schizophrenia, Human behaviour

## Abstract

Intrinsic motivation deficits are a prominent feature of schizophrenia that substantially impacts functional outcome. This study used cluster analysis of innate real-world behaviours captured during two open-field tasks to dimensionally examine heterogeneity in intrinsic motivation in schizophrenia patients (SZ) and healthy controls (HC). Wireless motion capture quantified participants’ behaviours aligning with distinct aspects of intrinsic motivation: exploratory behaviour and effortful activity in the absence of external incentive. Cluster analysis of task-derived measures identified behaviourally differentiable subgroups, which were compared across standard clinical measures of general amotivation, cognition, and community functioning. Among 45 SZ and 47 HC participants, three clusters with characteristically different behavioural phenotypes emerged: low exploration (20 SZ, 19 HC), low activity (15 SZ, 8 HC), and high exploration/activity (10 SZ, 20 HC). Low performance in either dimension corresponded with similar increased amotivation. Within-cluster discrepancies emerged for amotivation (SZ > HC) within the low exploration and high performance clusters, and for functioning (SZ < HC) within all clusters, increasing from high performance to low activity to low exploration. Objective multidimensional characterization thus revealed divergent behavioural expression of intrinsic motivation deficits that may be conflated by summary clinical measures of motivation and overlooked by unidimensional evaluation. Deficits in either aspect may hinder general motivation and functioning particularly in SZ. Multidimensional phenotyping may help guide personalized remediation by discriminating between intrinsic motivation impairments that require amelioration versus unimpaired tendencies that may facilitate remediation.

## Introduction

Motivation deficits are a prominent negative symptom of schizophrenia^1,2^ and are linked with patients’ community functioning^3–6^. Motivation in schizophrenia is typically assessed using interview-based rating scales^7^. The complexity of human motivation has, however, encouraged the development of objective tasks to quantify specific facets of motivation, primarily related to reward dysfunction^8–11^.

Previous investigations have largely focused on extrinsic motivation (i.e., behaviour instrumental to separable or external consequences, such as pursuing rewards or avoiding punishment). However, human behaviour is often intrinsically motivated (i.e., governed by inherent satisfaction associated with the behaviour itself rather than some separable consequence)^12^. Intrinsic motivation specifically has been associated with functional outcome^13–15^ and identified as an important treatment target in schizophrenia^16–18^. Intrinsic motivation has typically been assessed using rating scales^13,14,19^ or self-report questionnaires^15,20,21^. Task-based investigations have relied on self-reports of intrinsic motivation to engage in specific cognitive tasks^16,17,19,20^. This approach falls short of more direct measurement afforded by “free-choice” paradigms^22^, and may be problematic, particularly in individuals with schizophrenia, whose task-specific versus generalized intrinsic motivation appear to be divergent^19^. Further, in other fields such as educational psychology^23–25^ and computational neuroscience and neurorobotics^26,27^, intrinsic motivation has been conceptualized as a multidimensional construct (e.g., “knowledge-based” intrinsic motivation associated with learning, exploration, and novelty versus “competence-based” intrinsic motivation associated with affecting the environment and achieving self-determined goals).

We recently developed and preliminarily validated two tasks that evaluate intrinsically motivated behaviours. The Novelty Exploration Task quantifies exploratory behaviour in an unfamiliar setting containing common and uncommon objects^28^. The Activity Preference Task quantifies activity engagement under the provision of explicit choice between active versus passive engagement options^29^. Both tasks objectively measure real-world behaviour using wireless motion capture in an open-field setting, and neither incorporate extrinsic incentives (i.e., specific goals or rewards). We envisaged that Novelty Exploration Task behaviour (i.e., free exploration of a novel environment and stimuli) would align with knowledge-based intrinsic motivation, while Activity Preference Task behaviour (i.e., physical motion-based game engagement as a means of affecting the environment) would align with competence-based intrinsic motivation. In preliminary investigations, schizophrenia participants demonstrated somewhat diminished visual object exploration, but comparable active engagement as healthy control counterparts. Correlations between measures from both tasks and schizophrenia participants’ negative symptoms, amotivation, or community functioning supported task validity, indicating that task-assessed behavioural aberrations share common elements with wide-ranging motivation and functioning deficits.

Based on the premise that the Novelty Exploration and Activity Preference Tasks measure theoretically distinct dimensions of intrinsic motivation, we aimed to concomitantly examine performance across these tasks in schizophrenia and healthy control participants, and investigate the presence of subgroups with distinct behavioural phenotypes. We hypothesized that a multidimensional examination of intrinsic motivation would reveal task performance-based subgroups that did not strictly adhere to diagnostic boundaries, and with differential clinical and functional profiles. As a secondary aim of this study, we evaluated diagnostic group differences in task performance and clinical correlates of task performance to corroborate and extend the findings from the initial task validation studies.

## Results

Demographic and clinical characteristics of the 45 participants with schizophrenia or schizoaffective disorder (SZ) and 47 healthy control participants (HC) of this study are summarized in Table 1. This sample includes 41 participants who were assessed during the pilot-phase validation of the Novelty Exploration and Activity Preference Tasks^28,29^.

**Table 1:** Characteristics of the study sample.

	SZ (*n* = 45) median (*Q*_*1*_, *Q*_*3*_)	HC (*n* = 47) median (*Q*_*1*_, *Q*_*3*_)	Wilcoxon–Mann–Whitney / Fisher’s Exact Test *P*
Age,^a^ years	32.00 (26.00, 47.00)	34.00 (24.00, 46.00)	0.639
Sex,^a^ n_female_ : n_male_	17 (38%) : 28 (62%)	19 (40%) : 28 (60%)	0.833
Dx,^b^ n_Sz_ : n_SzAf_	40 (89%) : 5 (11%)		
Illness Duration,^b^ years	8.00 (5.00, 16.50)		
CPZ Equivalents,^b^ mg	450.00 (300.00, 641.43)		
SAPS^b^	5.00 (0.00, 16.00)		
SANS^b^	16.00 (9.50, 25.50)		
SANS-DimExp^b^	3.00 (1.00, 9.50)		
SANS-Amot^b^	12.00 (7.50, 18.00)		
CDSS^b^	1.00 (0.00, 3.00)		
SAS^b^	1.00 (0.00, 2.50)		
AES^c^	33.00 (27.50, 38.00)	24.00 (22.00, 25.00)	<0.001
BACS Z-score^c^	−1.12 (−1.78, −0.44)	0.47 (−0.33, 1.06)	<0.001
PSP^c^	55.00 (41.00, 65.00)	80.00 (75.00, 85.00)	<0.001
SFS^c^	113.57 (106.14, 117.39)	122.57 (119.43, 125.79)	<0.001
TEPS-Ant^d^	46.00 (38.00, 50.00)	44.00 (40.75, 50.25)	0.837
TEPS-Con^d^	38.00 (32.25, 41.00)	39.00 (35.75, 44.00)	0.038
DPB^d^	41.00 (26.00, 56.00)	34.00 (24.00, 43.00)	0.195
BIS^d^	63.50 (56.25, 71.25)	53.00 (47.00, 58.00)	<0.001
BAI^d^	8.00 (4.00, 13.75)	2.00 (0.00, 5.00)	<0.001
BFI-Extraversion^d^	23.00 (17.25, 27.75)	27.00 (24.00, 29.00)	0.079
BFI-Agreeableness^d^	36.50 (33.00, 40.00)	39.00 (33.00, 43.00)	0.182
BFI-Conscientiousness^d^	30.50 (28.00, 35.00)	37.00 (32.00, 42.00)	0.002
BFI-Neuroticism^d^	23.50 (18.25, 28.25)	20.00 (10.00, 23.00)	0.005
BFI-Openness^d^	36.00 (33.25, 41.00)	35.00 (33.00, 43.00)	0.855

SZ schizophrenia group, HC healthy control group.

^a^Case-control matching measures.

^b^Schizophrenia-specific clinical measures: Dx, diagnosis (Sz, schizophrenia; SzAf, schizoaffective disorder); CPZ, chlorpromazine dose; SAPS, Scales for the Assessment of Positive Symptoms; SANS, Scales for the Assessment of Negative Symptoms (DimExp, Diminished Expression subdomain; Amot, Amotivation subdomain); CDSS, Calgary Depression Scale for Schizophrenia; SAS, Simpson Angus Rating Scale.

^c^Primary clinical measures: AES, Apathy Evaluation Scale; BACS, Brief Assessment of Cognition in Schizophrenia; PSP, Personal and Social Performance Scale; SFS, Social Functioning Scale.

^d^Secondary/Exploratory measures (administered to subsamples): TEPS, Temporal Experience of Pleasure Scale (Ant, Anticipatory; Con, Consummatory); DPB, defeatist performance beliefs; BIS, Barratt Impulsiveness Scale; BAI, Beck Anxiety Inventory; BFI, Big Five Inventory.

### Task performance across diagnostic groups

Novelty Exploration and Activity Preference Task performance scores across diagnostic groups, alongside measures considered to be potential task-specific confounders or self-report-based validators, are summarized in Table 2.

**Table 2:** Behavioural task performance and task-related measures across diagnostic groups.

	SZ (*n* = 45) median (*Q*_*1*_, *Q*_*3*_)	HC (*n* = 47) median (*Q*_*1*_, *Q*_*3*_)	Wilcoxon–Mann–Whitney Test *P*
NET Distance^a^	95.24 (55.89, 177.66)	103.34 (47.31, 198.40)	0.988
NET Spatial *d*^a^	1.32 (1.25, 1.41)	1.29 (1.24, 1.38)	0.338
NET VisObjExp Count^a^	6.00 (4.25, 8.00)	8.00 (5.00, 9.00)	0.063
NET VisObjExp Time^a^	95.02 (24.24, 156.84)	102.69 (61.27, 183.05)	0.195
NET TacObjExp Count^a^	1.00 (0.00, 2.00)	1.00 (0.00, 2.00)	0.620
NET TacObjExp Time^a^	3.10 (0.00, 11.74)	6.19 (0.00, 30.13)	0.284
APT Active Time^b^	525.50 (213.75, 758.25)	637.00 (295.00, 869.00)	0.239
APT Switches^b^	10.00 (3.25, 16.75)	6.00 (2.00, 12.00)	0.073
APT Active Intensity^b^	1.04 (0.64, 1.46)	1.54 (0.98, 1.77)	<0.001
APT Active Persistence^b^	3.20 (2.45, 4.41)	4.05 (3.30, 4.85)	0.059
Pre-task Fatigue^c,d^	1.00 (0.00, 1.00)	1.00 (0.00, 1.00)	0.148
Post-task Fatigue^d^	1.00 (0.00, 1.00)	1.00 (0.00, 1.00)	0.884
NET Object Novelty^c^	13.00 (9.50, 16.50)	10.00 (6.00, 14.00)	0.024
NET Object Interest^c^	15.00 (10.00, 19.00)	13.00 (7.00, 15.00)	0.025
NET Anxiety Magnitude^c,e^	24.50 (5.25, 62.50)	6.00 (1.00, 13.00)	0.026
NET Anxiety Duration^c.e^	21.00 (4.25, 57.75)	6.00 (1.00, 9.00)	0.017
APT Anxiety Level^d,e^	8.00 (1.00, 35.25)	3.00 (0.00, 7.00)	0.057
APT Interest Level^d,e^	63.50 (38.75, 80.75)	67.00 (29.00, 77.00)	0.892
Finger Tapping Task^d,e^	51.60 (47.00, 57.80)	57.15 (52.20, 63.20)	0.005

SZ schizophrenia group, HC healthy control group.

^a^Novelty Exploration Task (NET) behavioural measures: Distance, total distance travelled (m); Spatial *d*, index of complexity of locomotion – lower values indicate more linear, less circumscribed movement; VisObjExp, visual object exploration (Count, number of objects explored; Time, duration (s) of exploration); TacObjExp, tactile object exploration (Count, number of objects explored; Time, duration (s) of exploration).

^b^Activity Preference Task (APT) behavioural measures: Active Time, duration (s) on active engagement option; Switches, number of switches between active and passive engagement options; Active Intensity, average hand speed (m/s) during periods of active engagement; Active Persistence, index of tendency to sustain continuous active engagement.

^c^NET-related measures.

^d^APT-related measures.

^e^Exploratory measures (administered to subsamples).

Omnibus group effects in permutation MANCOVA tests (producing empirical *P*-values, *P*_perm_) that compared Novelty Exploration Task performance between SZ and HC participants were nonsignificant for locomotion (*V* = 0.062, *F*(2,87) = 2.86, *P* = 0.062, *P*_perm_ = 0.060) and tactile object exploration (*V* = 0.030, *F*(2,86) = 1.33, *P* = 0.269, *P*_perm_ = 0.269), but significant for visual object exploration (*V* = 0.099, *F*(2,86) = 4.70, *P* = 0.012, *P*_perm_ = 0.010). Follow-up univariate tests determined that SZ participants visually explored significantly fewer objects (*F*(1,87) = 9.05, *P* = 0.003, *η*_p_^2^ = 0.094, *P*_perm_ = 0.003), though with nonsignificant reduction in duration of visual object exploration (*F*(1,87) = 3.52, *P* = 0.064, *η*_p_^2^ = 0.039, *P*_perm_ = 0.062). These findings remained unchanged after repeat MANCOVA tests that included age as an additional covariate due to significant correlations between Novelty Exploration Task performance and age, particularly in the SZ sample (Supplementary Table S2).

The Activity Preference Task performance MANOVA test revealed a significant omnibus diagnostic group effect (*V* = 0.138, *F*(4,86) = 3.44, *P* = 0.012, *P*_perm_ = 0.011). In follow-up univariate tests there was no significant group difference in active engagement duration (*F*(1,89) = 1.50, *P* = 0.225, *η*^2^ = 0.017, *P*_perm_ = 0.225) or persistence (*F*(1,89) = 2.00, *P* = 0.161, *η*^2^ = 0.022, *P*_perm_ = 0.166). SZ participants, however, demonstrated significantly diminished active engagement intensity (*F*(1,89) = 10.82, *P* = 0.001, *η*^2^ = 0.108, *P*_perm_ = 0.002), and nonsignificant increase in frequency of switching between activity options (*F*(1,89) = 3.55, *P* = 0.063, *η*^2^ = 0.038, *P*_perm_ = 0.061).

Between-group differences in pre-task and post-task fatigue were nonsignificant, with most participants indicating “None” (0) or “Mild” (1) fatigue (Table 2). Pre-task fatigue was not correlated with Novelty Exploration Task performance, and neither pre-task nor post-task fatigue was correlated with Activity Preference Task performance (Supplementary Table S1). The Novelty Exploration Task objects were collectively rated as being significantly more novel and more interesting by the SZ group compared to the HC group (Table 2). Object novelty score was significantly correlated with locomotion measures in the overall sample (Supplementary Table S1), but with no measure in the SZ sample (Supplementary Table S2). Object interest score was significantly correlated with several locomotion and object exploration measures in the overall sample (Supplementary Table S1) and in the SZ sample (Supplementary Table S2). A subsample of participants (*n*_SZ_ = 39, *n*_HC_ = 42) completed a Finger Tapping Task that was used to assess motor functioning in relation to Activity Preference Task performance. SZ participants demonstrated significantly worse finger tapping performance compared to HC participants (Table 2). Further, finger tapping performance was significantly correlated with the intensity of active engagement (i.e., effort exertion) in the overall sample (Supplementary Table S1), but with no measure in the SZ sample (Supplementary Table S2).

SZ participants reported feeling significantly more anxious during the Novelty Exploration Task and for a longer duration compared to HC participants (Table 2), although this was only evaluated in a subsample (*n*_SZ_ = 24, *n*_HC_ = 27). Anxiety magnitude was not significantly correlated with any task measure, and anxiety duration was significantly correlated only with visual object exploration duration (Supplementary Table S1). SZ participants in this subsample also reported experiencing greater anxiety than their HC counterparts during the Activity Preference Task, but to a nonsignificant extent (Table 2). These anxiety ratings were not significantly correlated with any task measure (Supplementary Table S1). This subsample of participants also indicated their level of interest in the Activity Preference Task, which did not differ significantly between diagnostic groups (Table 2), but was significantly correlated with active engagement duration and persistence (Supplementary Table S1).

### Clinical correlates of task performance

Spearman correlations between task performance and clinical characteristics were evaluated in the overall and SZ samples (Supplementary Tables S1 and S2, respectively). In the overall sample, visual object exploration in the Novelty Exploration Task and active engagement duration, intensity, and persistence in the Activity Preference Task were significantly correlated negatively with general amotivation measured by the Apathy Evaluation Scale (AES) and positively with community functioning measured by the clinician-rated Personal and Social Performance Scale (PSP) or the self-report Social Functioning Scale (SFS). Functioning (PSP) was also correlated with more linear (less circumscribed) locomotion pattern in the Novelty Exploration Task (lower spatial *d*). These relationships were largely consistent with trends within the SZ sample. SZ participants’ Novelty Exploration Task locomotion (including lower spatial *d* reflective of more linear locomotion), visual object exploration, and tactile object exploration as well as Activity Preference Task active engagement duration and intensity and activity switching frequency were significantly correlated negatively with general amotivation (AES or Scales for the Assessment of Negative Symptoms (SANS) Amotivation subdomain score). Most of these Novelty Exploration Task measures and Activity Preference Task active engagement intensity were also correlated significantly with SZ participants’ functioning (PSP or SFS). Further, consummatory pleasure measured by the Temporal Experience of Pleasure Scale (TEPS-Con) was correlated with Novelty Exploration Task tactile object exploration measures in the overall and SZ samples, and also with Activity Preference Task active engagement duration in the overall sample.

In the overall sample, cognition measured by the Brief Assessment of Cognition in Schizophrenia (BACS) was correlated with visual exploration object count and active engagement intensity. Among the secondary measures administered to a subsample (*n*_SZ_ = 24, *n*_HC_ = 27), extraversion measured by the Big Five Inventory (BFI) was correlated with Novelty Exploration Task locomotion (specifically, higher distance and lower (more linear) spatial *d*). Extraversion was also correlated with higher activity switching and lower active engagement persistence in the Activity Preference Task, which were also correlated with agreeableness (BFI) but in the opposite direction. Active engagement intensity was positively correlated with conscientiousness (BFI), and negatively correlated with impulsivity measured by the Barratt Impulsiveness Scale (BIS). In the SZ sample, cognition (BACS) was correlated with higher activity switching, and extrapyramidal symptom severity (Simpson Angus Rating Scale (SAS)) was correlated with lower active engagement intensity in the Activity Preference Task. Further, consistent associations emerged between task performance and age, illness duration, and antipsychotic medication dosage (chlorpromazine equivalents) in the SZ sample. Longer illness duration was significantly correlated with lower exploration across all Novelty Exploration Task measures, and SZ participant age was similarly correlated with most Novelty Exploration Task measures (notably, age was also correlated, to a lesser extent, with a subset of exploration measures and with lower activity switching in the overall sample). Higher medication dosage was significantly correlated with lower visual exploration object count and tactile object exploration in the Novelty Exploration Task, and with lower active engagement duration and intensity in the Activity Preference Task.

Due to the prominence of these relationships, particularly in the SZ sample, we further investigated whether these clinical measures were uniquely related to task performance, beyond coincidental relationship with amotivation in SZ measured by the AES. Most of the significant correlations involving clinical measures associated in some degree with motivation (i.e., negative symptoms and amotivation evaluated by the SANS, and community functioning evaluated by the PSP or SFS), as well as several other clinical measures (e.g., medication dosage), were nonsignificant in partial correlations controlled for the AES, implicating overlapping covariance with AES-quantified amotivation. Notable exceptions to this (demarcated within Supplementary Table S2) for the Novelty Exploration Task include significant partial correlations between tactile object exploration and consummatory pleasure (TEPS-Con). Further, several of the consistent correlations between task measures and illness duration (and, to a lesser extent, age) persisted after controlling for the AES, indicating a curious relationship between SZ participants’ illness chronicity and exploratory behaviour deficits independent of amotivation. Also, extrapyramidal side-effects (SAS) remained significantly correlated with Activity Preference Task active engagement intensity, which was unexpected due to the exclusion of participants with elevated SAS scores. Importantly, though, this relationship does not compellingly implicate amotivation-independent medication-induced motor deficits as an effector of task performance, considering that activity engagement intensity’s AES-controlled partial correlation with medication dosage and zero-order correlation with finger tapping performance were both nonsignificant.

### Clustering-based behavioural phenotypes

**Fig. 1:**
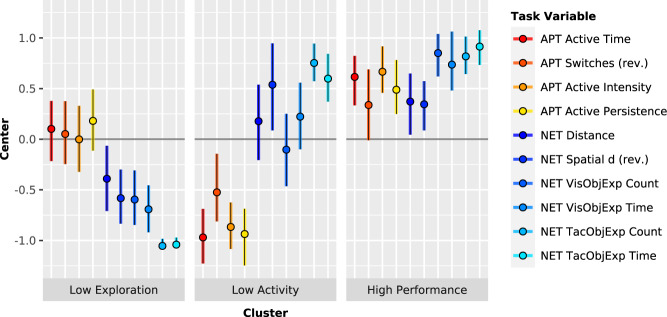
Cluster centers of the three-cluster sparse *k*-means solution described by behavioural task performance. Cluster-wise means and bootstrapped (bias-corrected and accelerated) 95% confidence intervals are shown for the clustering input variables, derived from the Activity Preference Task (APT) and Novelty Exploration Task (NET). All variables are shown on a common scale such that higher values indicate increasing performance, with appropriate variables being reverse scaled (rev.). Based on their characteristic low NET (with medium APT) performance, low APT (with medium NET) performance, and high APT and NET performance, we respectively refer to the clusters as Low Exploration, Low Activity, and High Performance. An alternative visualization of these data for cluster-wise comparison of individual clustering input variables is provided in Supplementary Fig. S1. Task variable (sparse *k*-means weight): APT Active Time (0.27), duration on active engagement option; Switches (0.07), number of switches between active and passive engagement options, reversed; Active Intensity (0.25), average hand speed during periods of active engagement; Active Persistence (0.25), index of tendency to sustain continuous active engagement; NET Distance (0.09), total distance travelled; Spatial *d* (0.17), index of complexity of locomotion, reversed; VisObjExp Count (0.26), number of objects visually explored; VisObjExp Time (0.27), duration of visual object exploration; TacObjExp Count (0.56), number of objects physically explored; TacObjExp Time (0.55), duration of physical object exploration.

Behavioural phenotypes of intrinsic motivation based on Novelty Exploration and Activity Preference Task performance were identified using sparse *k*-means clustering^30,31^, which combines input variable weighting with partitioning. The optimal number of partitions was determined to be *k* = 3 by the concurrence of three data-driven methods^32–34^. The three-cluster solution comprised clusters of 39 (42%), 23 (25%), and 30 (33%) participants, with non-zero weights for all task-derived input variables. Based on the clusters’ centers (Fig. 1), we refer to them as the Low Exploration (*n*_SZ_ = 20 (51%), *n*_HC_ = 19 (49%)), Low Activity (*n*_SZ_ = 15 (65%), *n*_HC_ = 8 (35%)), and High Performance cluster (*n*_SZ_ = 10 (33%), *n*_HC_ = 20 (67%)), respectively. Of note, locomotion complexity (Spatial *d*) and activity switching (Switches) were reverse scaled to simplify visualization, in keeping with clustering-based findings that higher values for these two performance measures corresponded to reductions in exploratory behaviour and activity engagement, respectively. Bootstrapped cluster-wise stability assessment^35^ revealed mean Jaccard similarity values of 0.915, 0.782, and 0.871 for the Low Exploration, Low Activity, and High Performance cluster, respectively, indicating that all three clusters were acceptably stable (>0.75) and two were highly stable (>0.85).

### Behavioural differences across clusters

Bootstrapped one-way ANOVA tests (producing empirical *P*-values, *P*_boot_), and post-hoc pairwise mean differences (∆mean) and 95% confidence intervals (CI), confirmed that the task-derived variables used to identify behavioural phenotypes discriminated subgroups that did indeed differ behaviourally to statistically significant extents (Supplementary Table S3). Omnibus cluster effects for all 10 Novelty Exploration and Activity Preference Task measures were significant at the uncorrected α = 0.05 threshold. The effects for the following Novelty Exploration Task measures remained significant after Bonferroni correction (demarcated in Supplementary Table S3): complexity of locomotion (Spatial *d*); number of objects visually explored and duration of visual object exploration (VisObjExp Count and Time); and number of objects physically explored (TacObjExp Count). The effects for the following Activity Preference Task measures were significant after correction (demarcated in Supplementary Table S3): duration, intensity (i.e., effort exertion), and persistence of active engagement (Active Time, Intensity, and Persistence).

The post-hoc pairwise contrasts across clusters for the task measures (Supplementary Table S3) further confirmed that certain behavioural deficits were specific to the Low Exploration or Low Activity cluster. Low Exploration participants’ exploratory behaviour was characterized by less linear locomotion and reduced visual and tactile exploration, and most pairwise contrasts versus Low Activity and versus High Performance participants reflecting these deficits remained significant (non-zero) following Bonferroni correction (i.e., the LE–LA and LE–HP contrasts for Spatial *d*, VisObjExp Time, and TacObjExp Count and Time, as well as the LE–HP contrasts for Distance and VisObjExp Count; Supplementary Table S3). Low Exploration participants’ activity engagement, however, was not so substantially diminished, as all Activity Preference Task measures placed these participants in the intermediate between Low Activity and High Performance participants; indeed, while Low Exploration participants demonstrated lower active engagement duration and intensity compared to High Performance participants (i.e., the LE–HP contrasts for Active Time and Intensity were significant following Bonferroni correction; Supplementary Table S3), activity switches and active engagement persistence did not differ significantly between these two clusters. Low Activity participants’ activity engagement was characterized by reduced active engagement duration, intensity, and persistence and increased activity switches, and most pairwise contrasts versus Low Exploration and versus High Performance participants for all Activity Preference Task measures remained significant following Bonferroni correction (i.e., the LE–LA and LA–HP contrasts for Active Time, Intensity, and Persistence, as well as the LA–HP contrast for Switches; Supplementary Table S3). For exploratory behaviour, however, Low Activity participants were intermediate between Low Exploration and High Performance participants for most measures, with only visual exploration reduced in the Low Activity compared to the High Performance cluster (i.e., the LA–HP contrasts for VisObjExp Count and Time were significant after Bonferroni correction; Supplementary Table S3).

Bootstrapped two-way ANOVA tests of diagnostic group by cluster interaction effects, and post-hoc within-cluster SZ versus HC contrasts, evaluated inconsistencies in diagnostic group differences in task performance across clusters. Among all task measures, a significant interaction effect was detected solely for intensity of active engagement in the Activity Preference Task (*F*(2,85) = 6.45, *P* = 0.002, *P*_boot_ = 0.002); only the Low Exploration cluster SZ participants demonstrated lower intensity versus HC counterparts (∆mean = −0.64, CI_95%_ = (−1.00, −0.30)), a difference that remained significant (non-zero) following Bonferroni correction.

### Clinical characteristics across clusters

**Fig. 2:**
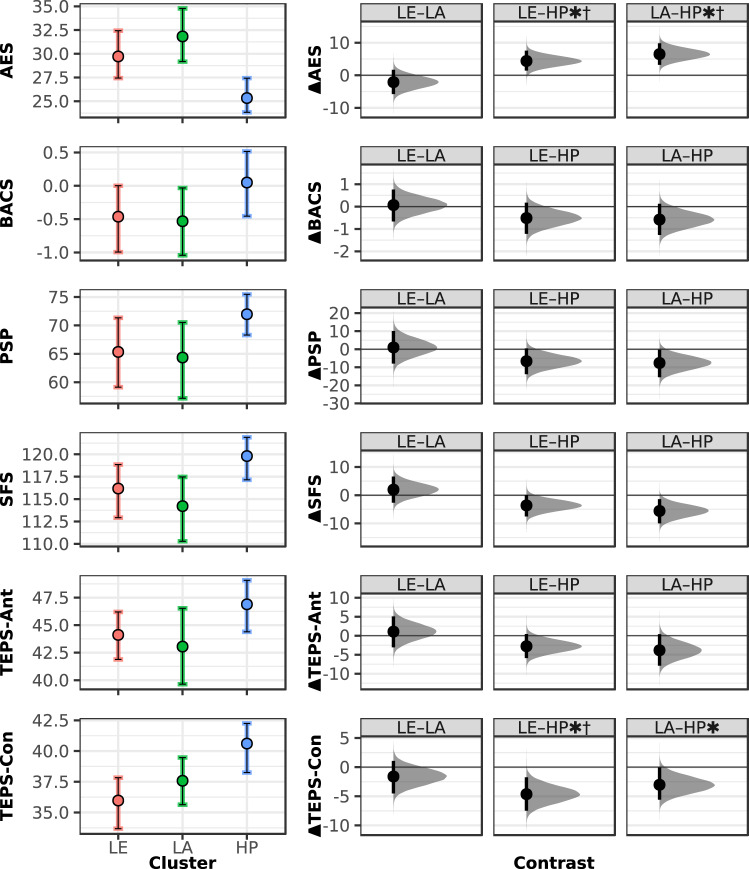
Clinical characteristics across clusters. Clinical profiles of the Low Exploration (LE), Low Activity (LA), and High Performance (HP) clusters (defined based on performance in the Activity Preference Task and Novelty Exploration Task) are shown for the (top to bottom) Apathy Evaluation Scale (AES), Brief Assessment of Cognition in Schizophrenia composite Z-score (BACS), Personal and Social Performance Scale (PSP), Social Functioning Scale (SFS), and Temporal Experience of Pleasure Scale Anticipatory (TEPS-Ant) and Consummatory subscales (TEPS-Con). Means and bootstrapped (bias-corrected and accelerated) 95% confidence intervals (CI) are shown for each cluster (left) and for differences between clusters in pairwise contrasts (right). Due to significant one-way omnibus cluster effects for the AES (*F*(2,89) = 6.31, *P* = 0.003, *P*_boot_ = 0.003) and TEPS-Con (*F*(2,79) = 5.35, *P* = 0.007, *P*_boot_ = 0.005), post-hoc pairwise contrasts across clusters for these measures were also evaluated for significance (* 95%, ^†^ Bonferroni-corrected 95% bootstrapped CI indicating significant (non-zero) mean difference). Numerical values of the visualized data and test statistics are provided in Supplementary Table S3.

The imbalance in allocation of SZ versus HC participants across the three clusters was nonsignificant (Fisher’s exact *P* = 0.067), but an exploratory comparison of High Performance (*n*_SZ_ = 10 (33%), *n*_HC_ = 20 (67%)) versus non-High Performance participants (*n*_SZ_ = 35 (56%), *n*_HC_ = 27 (44%)) indicated increased odds of diminished task performance in SZ participants (odds ratio = 2.57, CI_95%_ = (1.04, 6.79), *P* = 0.047). The clusters did not differ significantly in age or sex (Supplementary Table S3). Bootstrapped one-way ANOVA tests for cluster-wise comparisons of the primary clinical outcome measures (Fig. 2 and Supplementary Table S3) identified significant (Bonferroni-corrected) omnibus differences in the AES, but not in the BACS, PSP, and SFS. Omnibus differences also emerged in the TEPS-Con (Fig. 2 and Supplementary Table S3). AES differences were driven by higher scores in Low Exploration and Low Activity participants versus High Performance participants. TEPS-Con differences were similarly driven by elevated scores in High Performance participants. Given our focus on motivation deficits, we performed supplementary cluster-wise comparisons of the SANS in SZ participants that revealed a similar pattern of negative symptom severity and amotivation across clusters as seen with the AES in the full sample, albeit with nonsignificant omnibus effects in this small subsample for both the SANS total (*F*(2,42) = 2.40, *P* = 0.103, *P*_boot_ = 0.084) and SANS amotivation subdomain scores (*F*(2,42) = 2.04, *P* = 0.142, *P*_boot_ = 0.122). Further, given that age, illness duration, antipsychotic medication dosage, and extrapyramidal symptom severity had emerged as correlates of SZ participants’ task performance (Supplementary Table S2), we performed similar SZ-only comparisons for these measures, which revealed that omnibus differences were significant for age (*F*(2,42) = 3.64, *P* = 0.035, *P*_boot_ = 0.034) and medication dosage (*F*(2,42) = 4.13, *P* = 0.023, *P*_boot_ = 0.031), and marginally nonsignificant for illness duration (*F*(2,42) = 3.01, *P* = 0.060, *P*_boot_ = 0.056), but nonsignificant for extrapyramidal symptoms (*F*(2,42) = 2.03, *P* = 0.144, *P*_boot_ = 0.105), across clusters in the SZ sample.

Exploratory cluster-wise comparisons of other individual characteristics in a subsample of participants (Supplementary Table S3) identified significant omnibus differences in impulsivity (BIS; *F*(2,48) = 4.21, *P* = 0.021, *P*_boot_ = 0.020) and anxiety measured by the Beck Anxiety Inventory (BAI; *F*(2,48) = 6.96, *P* = 0.002, *P*_boot_ = 0.031), but not defeatist performance beliefs (DPB). Low Activity participants demonstrated significantly (Bonferroni-corrected) higher BIS scores versus High Performance participants, and higher BAI scores versus Low Exploration and versus High Performance participants. In the BFI, only conscientiousness differed significantly (*F*(2,48) = 4.82, *P* = 0.012, *P*_boot_ = 0.014), with High Performance participants scoring significantly (Bonferroni-corrected) higher versus Low Activity participants for this personality trait.

**Fig. 3:**
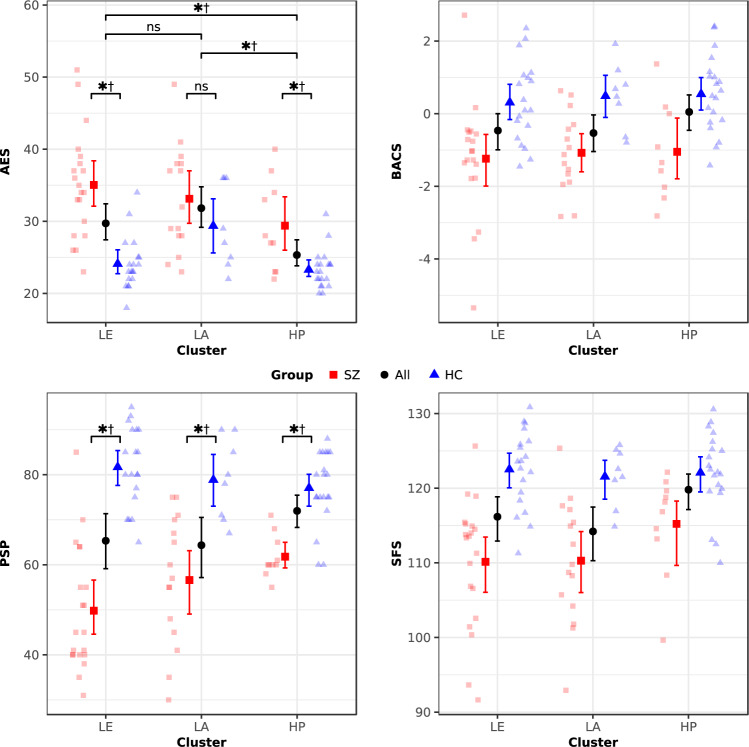
Amotivation, cognition, and functioning across clusters and diagnostic groups. Clinical profiles of schizophrenia (SZ) and healthy control participants (HC) across the Low Exploration (LE), Low Activity (LA), and High Performance (HP) clusters (defined based on performance in the Activity Preference Task and Novelty Exploration Task) are shown for the primary clinical measures: Apathy Evaluation Scale (AES, top-left), Brief Assessment of Cognition in Schizophrenia composite Z-score (BACS, top-right), Personal and Social Performance Scale (PSP, bottom-left), and Social Functioning Scale (SFS, bottom-right). Means and bootstrapped (bias-corrected and accelerated) 95% confidence intervals (CI) are shown for all within-cluster participants, and separately for SZ and HC participants; faded symbols represent individual participants. A significant one-way omnibus cluster effect for the AES (Fig. 2 and Supplementary Table S3) was followed by post-hoc pairwise contrasts across clusters (* 95%, ^†^ Bonferroni-corrected 95% bootstrapped CI indicating significant (non-zero) mean difference; ^ns^ zero-containing 95% CI indicating nonsignificant difference). Post-hoc within-cluster SZ versus HC contrasts were performed following evaluation of two-way omnibus diagnostic group by cluster interaction effects for the AES (*F*(2,86) = 3.12, *P* = 0.049, *P*_boot_ = 0.062) and PSP (*F*(2,86) = 4.86, *P* = 0.010, *P*_*boot*_ = 0.007), but not the BACS (*F*(2,84) = 0.00, *P* = 0.998, *P*_boot_ = 0.997) and SFS (*F*(2,86) = 1.36, *P* = 0.263, *P*_boot_ = 0.217). AES scores differed between groups within the LE (∆mean = 10.94, CI_95%_ = (7.55, 14.61)) and HP clusters (∆mean = 6.10, CI_95%_ = (2.45, 10.10)), but not the LA cluster (∆mean = 3.76, CI_95%_ = (−1.39, 9.12)). PSP scores were significantly lower for SZ participants within each cluster, with the discrepancy versus HC participants growing from the HP cluster (∆mean = −15.25, CI_95%_ = (−19.30, −10.25)) to the LA cluster (∆mean = −22.27, CI_95%_ = (−31.81, −13.55)) to the LE cluster (∆mean = −31.88, CI_95%_ = (−38.39, −24.16)).

Evaluation of diagnosis by cluster interactions for the primary clinical measures via bootstrapped two-way ANOVA tests (Fig. 3) identified a significant effect for clinician-rated community functioning. Within-cluster differences in SZ versus HC participants’ PSP scores increased from the High Performance cluster to the Low Activity cluster to the Low Exploration cluster. We also performed within-cluster group comparisons for general amotivation, having surmised that evaluating these contrasts directly would be preferable to simply assuming that between-group differences were consistent across clusters, especially considering the aforementioned significant between-cluster differences for this primary measure. Indeed, these post-hoc contrasts identified SZ versus HC inhomogeneity in AES scores within the Low Exploration and High Performance clusters, but not the Low Activity cluster.

### Cluster analysis of groups-separated data

We evaluated solutions produced by cluster analysis applied separately to the SZ and HC sample data (Supplementary Table S4) in order to explore whether the cluster structure identified in the overall sample data could also be identified in each diagnostic group independently. Specifically, we performed standard *k*-means clustering using as input group-separated data that were transformed by variable weights that had resulted from the above-described original sparse *k*-means cluster analysis.

For the SZ subsample (Supplementary Table S4), the two-cluster solution (deemed best by two of three methods used to determine the optimal number of clusters) comprised highly stable clusters and distinguished SZ participants with Low Exploration versus non-Low Exploration characteristics (i.e., most members had been classified as Low Activity or High Performance in the original solution). A three-cluster solution (evaluated for completeness, but not recommended by any method used to determine the optimal number of clusters) also produced two acceptably stable clusters demonstrating Low Exploration and non-Low Exploration characteristics, alongside another unstable/suspect cluster demonstrating Low Performance characteristics (i.e., all members had been classified as Low Exploration or Low Activity in the original solution). Thus, while the original three-cluster solution identified in the overall sample data did not appear to be reliably identifiable in the subset of these data with only SZ participants (comprising ~49% of the original sample size), there continued to be evidence of subgroups of SZ participants with differential task performance profiles.

For the HC subsample (Supplementary Table S4), the two-cluster solution (deemed best by two of three methods used to determine the optimal number of clusters, and deemed acceptable by the other) was similar to that for SZ, with highly stable clusters that distinguished HC participants with Low Exploration versus non-Low Exploration characteristics. The three-cluster solution (deemed best by one method used to determine the optimal number of clusters) also produced highly stable clusters. Further, these clusters aligned almost exactly with the Low Exploration, Low Activity, and High Performance clusters in original solution, with disagreement in the classification of only one participant. Thus, the original three-cluster solution identified in the overall sample data also appeared to be at least plausibly identifiable in the subset of these data with only HC participants.

### Cluster analysis of covariate-adjusted data

We performed a supplementary cluster analysis of task data after adjusting each performance measure by participant age, illness duration, and antipsychotic medication dosage. Compared to the original solution, the three-cluster solution for these covariate-adjusted data, deemed optimal by two of three *k* selection methods and representing the more parsimonious solution, comprised similarly sized stable clusters that corresponded roughly to the Low Exploration, Low Activity, and High Performance clusters, with disagreement in the classification of nine participants (Supplementary Table S5).

The statistical comparisons across the covariate-adjusted clusters were re-evaluated for the measures selected as covariates, which had differed in SZ participants across clusters in the original solution. The omnibus effects for age in the overall sample (*F*(2,89) = 0.18, *P* = 0.834, *P*_boot_ = 0.839) and the SZ sample (*F*(2,42) = 1.05, *P* = 0.358, *P*_boot_ = 0.370) were nonsignificant. The effects for illness duration (*F*(2,42) = 0.37, *P* = 0.692, *P*_boot_ = 0.704) and medication dosage (*F*(2,42) = 0.65, *P* = 0.525, *P*_boot_ = 0.524) in the SZ sample were also nonsignificant. These results collectively supported our expectation that clustering task data that was adjusted for these measures would redress cluster-wise differences in the original solution in these potential confounders.

The statistical cluster-wise comparisons were also re-evaluated for the task performance measures to ensure that behavioural differences in the original cluster solution had not been substantively nullified by covariate adjustment. As with the original solution, the omnibus effects in the overall sample for all task measures were significant (*P* ≤ 0.024, *P*_boot_ ≤ 0.044), and the effects, except for Activity Preference Task activity switches and Novelty Exploration Task locomotion extent and tactile object exploration duration, remained significant following Bonferroni correction (*P* < 0.001, *P*_boot_ < 0.001). Further, post-hoc pairwise contrasts generally confirmed the expected patterns of decreasing Novelty Exploration Task performance across the High Performance to Low Activity to Low Exploration cluster and decreasing Activity Preference Task performance across the High Performance to Low Exploration to Low Activity cluster. There were a few dissimilarities between the original versus covariate-adjusted solutions, however, with respect to which contrasts for these measures were (non)significant before and after Bonferroni correction.

Finally, we also re-tested our primary hypotheses regarding motivation and functioning in the covariate-adjusted cluster solution. As with the original clusters, the omnibus effect was significant (Bonferroni-corrected) for the AES in the overall sample (*F*(2,89) = 4.76, *P* = 0.011, *P*_boot_ = 0.012). However, while AES scores were higher in both the Low Exploration and Low Activity clusters compared to the High Performance cluster, only the Low Activity versus High Performance post-hoc contrast was significant with or without Bonferroni correction (∆mean = 6.01, CI_95%_ = (2.52, 9.52)). The omnibus effects remained nonsignificant for the BACS, PSP, and SFS.

## Discussion

The aim of this study was to identify behavioural phenotypes of intrinsic motivation in SZ and HC participants to test the hypothesis that those phenotypes would be clinically meaningful. We applied cluster analysis to real-world task performance data that quantified two dimensions of intrinsic motivation, which resulted in three clusters with significantly different behaviour. Comparison of clinical characteristics as a means of externally validating the clusters (i.e., determining their predictive validity with respect to variables not included in the clustering process) suggested that these phenotypes may be important clinical indicators, particularly for motivation deficits. Evaluation of the clusters’ clinical characteristics alongside diagnostic group status highlighted varying degrees of impairment specifically among SZ participant subgroups, which may further our understanding of motivation and functioning deficits that are central to schizophrenia. Further, our analyses of clinical correlates of intrinsically motivated exploratory behaviour and activity engagement, and diagnostic group differences in these behaviours, largely corroborated the findings of our preliminary task validation studies, which was a secondary aim of this study. These analyses also provide insights into how specific task behaviours may be influenced by differences in clinical characteristics across the clustering-based behavioural phenotypes.

The cluster solution demonstrated the multidimensional nature of intrinsic motivation. Non-zero weights for variables across both tasks supported our hypothesis that clustering-based behavioural phenotypes would be more discretely differentiated by multidimensional intrinsic motivation characterization, rather than by measurements derived from a specific task. High Performance participants’ behavioural characteristics indicated that intrinsically motivated participants performed well on both tasks. With regard to previously posited component-wise conceptualizations of intrinsic motivation^23–27^, this concordance possibly reflects integration of knowledge-based and competence-based intrinsic motivation towards driving innate behaviours. Further, this concordance appears to coincide with previous functional neuroimaging task-based identification of common neurobiological substrates for intrinsic motivation dimensions in nonclinical individuals^36^.

Conversely, Low Exploration and Low Activity participants’ discordant task performance indicates a behaviourally demonstrable disconnection between intrinsic motivation dimensions, or divergent orientation towards competence-based versus knowledge-based intrinsic motivation (as previously observed in nonclinical individuals^23^). Perhaps these discordances or divergences are more detectable in real-world behaviours or emerge more prominently in psychopathology-inclusive samples. Notably, the results of our supplementary cluster analysis of groups-separated data appear to support the former proposition, as the three-cluster structure comprising Low Exploration, Low Activity, and High Performance clusters was also stably identifiable in HC-only data. These analyses also highlighted, considering the absence of a similar three-cluster structure in SZ-only data, that dimensional evaluation of real-world performance (i.e., without imposing predetermined diagnostic boundaries) may benefit the identification of distinct behavioural deficits with potentially important implications for clinical psychopathology.

Cluster-wise comparisons of clinical measures demonstrated the cluster solution’s clinical relevance. Although both clinical amotivation and cognition differed between diagnostic groups, the task-based phenotypes reflected differences in only the former. This concurs with prior findings that Novelty Exploration and Activity Preference Task performance correlated with motivation deficits, but neither task appeared to pose substantial cognitive demand^28,29^. Our current secondary evaluation also largely reflected that task performance measures were correlated more substantially and consistently with amotivation than cognition. General motivation measured by the AES was least impaired in High Performance participants, whose intrinsic motivation appeared intact across dimensions; for these (mostly HC) participants, intrinsic motivation measured within the context of tasks designed to elicit specific behaviours corresponded with a broader range of motivated behaviours encompassed by the AES.

Low Exploration and Low Activity participants demonstrated similar increased AES scores compared to High Performance participants. This similarity suggests that analogous deficits in general motivation can emerge via different processes, including via predominant dysfunction in either knowledge-based or competence-based intrinsic motivation. Alternatively, behavioural expression of general impairment in motivation may differ across individuals, specifically resulting in reduced acquisition of knowledge about one’s surroundings or reduced environmental manipulation to suit innate objectives. Though incapable of resolving between these two proposed mechanisms, this investigation compellingly highlights that a summary clinical amotivation measure may conflate deficits that exhibit themselves rather differently in terms of outwardly observable behaviours.

In the High Performance and Low Exploration clusters, SZ participants demonstrated higher AES scores compared to within-cluster HC counterparts. The within-cluster differences potentially reflect deficits that the tasks were not designed to capture, namely extrinsic motivation deficits identified by investigations of reward-driven behaviour^8–11^. Although speculative in the absence of data that allows concurrent comparison of intrinsic versus extrinsic motivation, this proposition appears to be supported particularly by studies in schizophrenia that have consistently demonstrated associations between reduced reward-driven effort expenditure evaluated by effort-cost computation tasks and more severe negative symptoms or amotivation evaluated by clinical rating instruments^9,37–42^. It is plausible, therefore, that our primary clinical measure of amotivation may have captured, at least partially, schizophrenia-specific deficits that are more akin to extrinsic rather than intrinsic motivation deficits, and were thus not completely paralleled by intrinsically motivated task performance. Alternatively, similar intrinsically motivated tendencies in these SZ and HC participants may translate divergently to broadly defined motivated behaviour, perhaps implicating impaired willingness or capacity to leverage behavioural potential towards effective output in SZ participants. The latter proposition is partially supported by a task performance disparity specifically within the Low Exploration cluster—SZ participants demonstrated significantly lower active engagement intensity in the Activity Preference Task, indicating reduced effort exertion, versus HC counterparts, and this coincided with the largest within-cluster AES score discrepancy. Considered alongside previously reported associations between diminished reward-driven effort and amotivation^9,10,37,40–43^, our findings highlight effort dysfunction’s ubiquitous role in motivation in schizophrenia, beyond behaviour in pursuit of external rewards. Importantly, among some amotivated SZ participants this diminishment seems evident even compared to HC participants who demonstrate similar inclination towards effortful activity.

In contrast, Low Activity participants’ AES scores were similar across diagnostic groups, owing to HC participants’ elevated scores. Diminished competence-based intrinsic motivation, reflected by deficient activity engagement, may thus be a consistent predictor of generally reduced motivation to the exclusion of clinical diagnosis. Notably, anxiety and impulsivity were elevated in Low Activity participants, and in the SZ subsample compared to the HC subsample in general. While a substantial correlation between the BIS and active engagement intensity suggests that Low Activity participants’ increased impulsivity might be tied chiefly to their intrinsically motivated effort exertion deficits, no such relationship emerged between anxiety measured with the BAI and any task measure. Further, our supplementary analyses of task-specific anxiety in the same subsample revealed no significant relationship with any activity engagement measure (and a significant relationship with only a visual object exploration measure). Prior findings, however, have suggested that anxiety or neuroticism influence intrinsic motivation in schizophrenia^44,45^, and performance anxiety hinders engagement and enjoyment to the detriment of intrinsic motivation in nonclinical individuals^46,47^. Further investigation is thus warranted to elucidate relationships between these and other individual characteristics and competence-based intrinsic motivation deficits. Nonetheless, the Low Activity cluster’s skewed composition towards SZ participants potentially implicates schizophrenia-specific susceptibility to competence-based intrinsic motivation deficits. Indeed, previous investigations have stressed the importance of competency beliefs in schizophrenia for everyday goal setting^48^ and emotional experience^49^, alongside intrinsically motivated task performance^17,50^.

A more pronounced diagnosis by cluster interaction effect emerged for the PSP; SZ participants demonstrated progressively disparate clinician-rated functioning across clusters compared to the relative cluster-wise consistency of HC participants’ scores. A level of persistent within-cluster SZ versus HC discrepancy suggests that factors unrelated to the Novelty Exploration and Activity Preference Tasks (e.g., availability of opportunities, adequate social support, and abundance of extrinsic motivators) may allow HC participants to function sufficiently despite potential intrinsic motivation deficits. Greater disparity within the Low Exploration and Low Activity clusters compared to the High Performance cluster suggests that deficits in either intrinsic motivation dimension specifically in SZ participants correspond with further diminishment of functioning. The within-cluster PSP difference was greatest in the Low Exploration cluster, indicating a particular contribution of intrinsically motivated effort exertion towards SZ participants’ functioning. Overall, it appears that in addition to potential factors that are exogenous to intrinsic motivation, most SZ participants are encumbered by intrinsic motivation deficits, and some are impaired further by intrinsically motivated effort dysfunction, that pose additional hurdles to overcome for adequate community functioning. Our correlation analyses further supported this assertion, as PSP scores were associated with several Novelty Exploration Task measures in the overall and SZ samples and with several Activity Preference Task measures in the overall sample, but particularly with active engagement intensity among the Activity Preference Task measures in the SZ sample. Intrinsic motivation has previously been associated with functioning in schizophrenia^13–15^, and its malleability and central role in learning has deemed it a valuable psychosocial intervention target^17,18,51^. Multidimensional behavioural phenotyping may thus help guide personalized therapy by differentiating between intrinsic motivation impairments demanding amelioration versus unimpaired tendencies that may be leveraged to facilitate remediation.

The findings of this study should be interpreted in the context of several limitations, including the stimulus-contingent specificity of the tasks relative to the broad construct of intrinsic motivation. Namely, the Novelty Exploration Task objects and Activity Preference Task engagement options are presumed to represent common and uncommon objects, and active and passive activities, respectively, in general. With the current study, however, we are unable to confirm the generalizability of the task stimuli to other real-world stimuli, and this reflects the inherent challenge in designing ecologically valid behavioural tasks. Future studies that incorporate additional real-world metrics of intrinsic motivation represent an important next step in replicating and confirming the results from these tasks.

It is also possible that behaviours demonstrated by participants during the tasks were influenced by other factors beyond intrinsic motivation or typical clinical characteristics. Our analyses of individual characteristics (including personality traits, defeatist beliefs, anxiety, and impulsivity) and task-specific factors (including fatigue, anxiety, and motor functioning) indicated that certain individual characteristics may be related to particular task behaviours or particular behavioural phenotypes. Given the limited sample size and exploratory nature of these analyses, however, further investigation is required to ascertain whether these individual characteristics (or other characteristics that we had not considered or measured) impact task performance. Task performance may also be influenced by other factors including age, illness duration, or antipsychotic medication dosage. Our supplementary analyses of task data adjusted for these variables, however, identified a three-cluster solution similar to the original solution, with analogous cluster-wise differences in task performance and clinical amotivation. This supports our original assertion that the task performance-based clusters primarily reflect behavioural phenotypes of intrinsic motivation. That said, future studies of intrinsic motivation would ideally be designed to elucidate the nature of potential relationships with personality traits and other individual characteristics, as well as temporal relationships with illness progress, and potential effects of antipsychotic treatment.

The exploratory nature of cluster analysis may also be considered a limitation of this study; we employed data-driven, instead of hypotheses-driven, approaches to identifying the number and central characteristics of behavioural phenotypes. Replication of these findings in a larger independent sample is needed to confirm the behavioural phenotypes identified here, and potentially identify additional phenotypes that may have been absent in our sample—e.g., no cluster in our original solution demonstrated low performance on both tasks. Absence of such a subgroup may also be attributable to our focus on stable community-dwelling schizophrenia outpatients, precluding participation of individuals with the severest amotivation (i.e., prohibitive to voluntary research study participation). Beyond the identification of additional behavioural phenotypes, larger replication studies are also needed to address our current sample size limitations, especially given the substantial number and variety of measures administered and statistical tests conducted, and the reduced sample sizes that comprised the exploratory analyses. Considering that our SZ participants were stable outpatients treated with antipsychotic medication, our findings may not generalize to unmedicated or acutely psychotic patients, or inpatients with schizophrenia.

In summary, the Novelty Exploration and Activity Preference Tasks quantify aspects of intrinsic motivation and revealed distinct behavioural phenotypes. The clusters’ characteristics demonstrated that diminished intrinsically motivated exploratory tendencies or effortful engagement correspond to deficits in a wider range of motivated and functional behaviours. The prominence of this relationship particularly in our SZ participants shows the extent of intrinsic motivation deficits in schizophrenia and their broader impact. Multidimensional characterization of innate behaviour thus presents an interesting avenue of research for furthering our understanding of motivation deficits in schizophrenia. The application of the Novelty Exploration and Activity Preference Tasks to this end complements existing methods of quantifying extrinsic motivation, which may be particularly important given the lack of objective methods for assessing intrinsic motivation.

## Methods

### Participants

This case-control study included participants with schizophrenia or schizoaffective disorder (SZ) and healthy control participants (HC), group-matched for age and sex. All participants were aged 18–55 years, with no history of active substance abuse or dependence in the past three months (except for nicotine), or neurological disease. SZ participants were recruited from outpatient clinics and met the following inclusion criteria: DSM-IV diagnosis of schizophrenia or schizoaffective disorder, and no other concurrent Axis I disorder, using the Mini International Neuropsychiatric Inventory (MINI)^52^; stable dose of antipsychotic medications for at least the preceding four weeks; absence of akathisia (global item ≤2 on the Barnes Akathisia Rating Scale^53^); and absence of extrapyramidal symptoms (ratings ≤ 2 on ≤2 items on the Simpson Angus Rating Scale (SAS)^54^). HC participants did not meet criteria for any Axis I disorder, and had no family history of schizophrenia or related psychotic disorder in a first-degree relative. The study was undertaken at the Centre for Addiction and Mental Health. The study was approved by the Centre for Addiction and Mental Health Research Ethics Board. Participants were recruited from February 2013 to January 2018, provided written informed consent, and were compensated for participation.

### Clinical characterization

Participants were administered the Apathy Evaluation Scale – clinical version (AES)^55^, Brief Assessment of Cognition in Schizophrenia (BACS)^56^, Personal and Social Performance Scale (PSP)^57^, and Social Functioning Scale (SFS)^58^ as the primary clinical outcome measures of amotivation, cognition, and community functioning (clinician-rated and self-report), respectively. BACS composite Z-scores were determined using age and sex normative data. SFS global scores were computed as the mean of the seven subscales’ scaled scores.

SZ participants were also administered the Scales for the Assessment of Positive Symptoms and Negative Symptoms (SAPS and SANS)^59,60^, Calgary Depression Scale for Schizophrenia (CDSS)^61^, and SAS to measure symptom severity, depression, and medication-induced motor side-effects, respectively. SANS subdomain scores were computed for Diminished Expression (the sum of Affective Flattening subscale and Poverty of Speech items) and Amotivation (the sum of Avolition-Apathy and Anhedonia-Asociality subscale items)^62^. Chlorpromazine dose equivalents were calculated for SZ participants’ antipsychotic medications^63,64^.

The Temporal Experience of Pleasure Scale (TEPS)^65^ was administered to a subsample (*n*_SZ_ = 40, *n*_HC_ = 42) to measure anticipatory (TEPS-Ant) and consummatory pleasure (TEPS-Con). We also profiled a subsample’s (*n*_SZ_ = 24, *n*_HC_ = 27) personality traits using the Big Five Inventory (BFI)^66^, defeatist performance beliefs (DPB) using the DPB subscale of the Dysfunctional Attitudes Scale^67^, impulsivity using the Barratt Impulsiveness Scale (BIS)^68^, and anxiety using the Beck Anxiety Inventory (BAI)^69^.

### Behavioural characterization

Participants’ intrinsically motivated exploratory behaviour and activity engagement were characterized by the Novelty Exploration and Activity Preference Tasks that objectively quantified real-world behaviours in an open-field setting using wireless motion capture. Specific details pertaining to task design, data processing, and performance measures for each task have been published previously^28,29^, and the following are brief descriptions.

The Novelty Exploration Task evaluates exploratory behaviour in the presence of unfamiliar stimuli in an unfamiliar setting. Participants were asked to wait in a room that contained five commonplace and five uncommon objects, but no chair, for 15 min. The LIBERTY LATUS (Large Area Tracking Untethered System) (Polhemus Inc., Colchester, VT) was used to capture behaviour by tracking three participant-worn wireless markers’ positions and orientations: one on each wrist (estimating hand position), and one attached to a hat (estimating head position and orientation). Six performance measures were computed: extent and complexity of locomotion were indexed, respectively, by (1) distance travelled and (2) spatial *d*, a scaling exponent representing the geometric structure of a travelled path with values spanning 1 (long-range, straight movement) to 2 (local, highly circumscribed movement)^70,71^; visual object exploration was indexed by the (3) number of objects viewed and (4) total viewing duration, computed using head orientation; and tactile object exploration was indexed by the (5) number of objects physically inspected and (6) total object inspection duration, computed using head orientation and hand position.

The Activity Preference Task evaluates activity engagement under provision of an explicit choice between active versus passive engagement in the absence of external incentive. Participants had the options of watching a film seated (passive engagement) or playing a physical motion-based computer game (active engagement), at opposite ends of the room, while alone for 15 min. Participants could engage in either activity at any time, but the task duration was not disclosed, and no specific objective or incentive was implied. Task behaviour was captured in the same manner as for the Novelty Exploration Task. Proximity and orientation relative to the activities and hand motion were used to ascertain engagement in the active or passive option, or neither (or ambiguous). Four performance measures were computed: (1) duration spent on the active engagement option; (2) number of switches between the active and passive engagement options; (3) intensity of active engagement based on hand motion during active engagement; and an (4) index of persistence of active engagement based on exponential model fitting of uninterrupted active engagement periods.

Participants were administered several self-report measures to assess specific factors that could potentially affect Novelty Exploration and Activity Preference Task performance. Prior to and after completing the tasks, all participants indicated their current level of fatigue on a four-point Likert scale. Following task completion, participants also rated each Novelty Exploration Task object for level of novelty and interest on a five-point Likert scale, which were summed to compute a total object novelty score and a total object interest score, respectively. In a subsample of participants (*n*_SZ_ = 24, *n*_HC_ = 27), we also evaluated Novelty Exploration Task-specific anxiety using visual analogue scales to capture participants’ magnitude and duration of anxiety.

As the active engagement option in the Activity Preference Task required physical motion for engagement, we assessed motor functioning via a computerized version of the Finger Tapping Task that required repeated pressing of a keyboard key over 10-s trials (adopted from the Halstead-Reitan Neuropsychological Test Battery) in a subsample of participants (*n*_SZ_ = 39, *n*_HC_ = 42). Further, in a similar manner as for the Novelty Exploration Task, a subsample of participants (*n*_SZ_ = 24, *n*_HC_ = 27) rated their level of anxiety during the Activity Preference Task on a visual analogue scale. These participants likewise rated their level of interest in the Activity Preference Task, intended to be a self-report validator of the relationship between perceived interest and behavioural engagement in the Activity Preference Task.

### Missing data

Novelty Exploration Task head marker data were unavailable for one SZ participant (*n*_SZ_ = 44 for visual and tactile object exploration). Activity Preference Task data were unavailable for one SZ participant (*n*_SZ_ = 44). Further, one HC participant did not engage in the active option, and hence active engagement intensity was incalculable and persistence was not estimated by exponential modelling (theoretically, negative infinite). Active engagement persistence was also incalculable for another SZ participant who engaged only in the active option (theoretically, infinite). To avoid forfeiting these as missing observations, active engagement intensity was set to zero for the former participant, and active engagement persistence across the entire sample was adjusted using 95% winsorization. The following data were also unavailable: BACS composite Z-scores for two SZ participants (*n*_SZ_ = 43), and DPB for one SZ participant (*n*_SZ_ = 23).

### Cluster analysis

All analyses were conducted using R version 3.3.2^72^.

#### Task data preparation

In order to identify behavioural phenotypes of intrinsic motivation based on Novelty Exploration and Activity Preference Task performance, we applied cluster analysis to the 10 task-derived behavioural variables. Each variable underwent Box-Cox power transformation (or Yeo-Johnston power transformation for variables without strictly positive values) using the R package “car” version 2.1–5^73^, and was subsequently centered to mean and scaled by standard deviation.

#### Sparse *k*-means clustering

Clustering was performed using the sparse *k*-means clustering algorithm^30^ implemented in the R package “RSKC” version 2.4.2^31^. This procedure combines automated selection and weighting of input variables with partitioning, producing a solution wherein cluster separation is maximized by optimally reweighting the squared Euclidean dissimilarities^30^. The following parameters were specified in addition to the input data: (1) the number of clusters, *k*; (2) a tuning parameter, *l*, that determines the degree of sparsity by constraining variable weights; (3) the proportion of observations to be trimmed, *α*, to provide a robust alternative to sparse *k*-means clustering; and (4) the number of random initial sets of cluster centers to be evaluated as staring points at each *k*-means partitioning step.

We aimed to identify the optimal number of clusters in the range *k* ∈ [2..10] based on three methods that compare cluster solutions obtained using the observed data versus those obtained using reference datasets (generated under a null distribution with *k* = 1): the Clest method^32^, the gap statistic^33^, and the weighted gap statistic^34^. Reference data for all three methods were generated from a uniform distribution with a box aligned with the principal components of the data^33^.

We applied Clest using a modified version of its implementation for sparse *k*-means clustering in the RSKC package, and selected *k* based on an adjusted Clest statistic (i.e., largest favourable difference between classification error rates for random partitions of the observed data versus classification error rates for random partitions of a collection of reference datasets, scaled by the standard deviation of the latter) and statistical significance (i.e., *P* < 0.05 against the null hypothesis that *k* = 1). For our observed data classification error rates, the dataset was randomly partitioned 2000-fold. For our reference data classification error rates, 2000 reference datasets were randomly generated and each was randomly partitioned 500-fold.

We evaluated the gap and weighted gap statistics (using a modified version of the implementation of gap statistic calculation in the R package “cluster” version 2.0.6) also based on 2000 randomly generated reference datasets, and selected the number of clusters using a “1-standard-error” style rule: the smallest *k* with a gap value greater than the gap value of *k* + 1 minus one standard error of gap of *k* + 1^33^. To ensure that our adaptation of these calculations for sparse *k*-means clustering did not result in misleading conclusions regarding selection of the optimal *k*, we also evaluated the gap and weighted gap statistics using standard *k*-means clustering (using the RSKC package with the sparsity parameter *l* modified accordingly) after transforming the input data with variable weights selected by sparse *k*-means clustering.

We specified the sparsity parameter *l* to a value that would not constrain variable weights, as we had no a priori requirement for which or how many task variables should be selected, while still anticipating that individual task variables may bear different degrees of utility (different weights) for clustering. We also confirmed that our choice of a non-weight-constraining *l* for the optimal *k* was appropriate using a data-driven approach (implemented in the R package “sparcl” version 1.0.4)^30^ that involved 2000 random permutations of (1) a dataset with only complete cases and (2) a dataset with missing values imputed (based on distance-weighted averages of the incomplete cases’ five nearest neighbours, using the R package “DMwR2” version 0.0.2).

We specified the observation trimming parameter to *α* = 0—with no trimming, the clustering performed was sparse *k*-means rather than robust and sparse *k*-means clustering. And finally, we specified that 1000 random initial sets of cluster centers should be evaluated as staring points at each *k*-means partitioning step.

#### Bootstrapped cluster stability and validity assessment

We performed resampling-based cluster-wise stability assessment using the R package “fpc” version 2.1–11.1^35^. We specifically evaluated the mean Jaccard similarities between clusters in the original solution versus the most similar clusters in 2000 bootstrapped samples (omitting multiple points) based on the criteria that valid, stable clusters should yield Jaccard values >0.75 and highly stable clusters should yield values >0.85.

#### Supplementary evaluation of alternative cluster solutions

We further explored potential cluster structures in the task data using two alternative approaches: (1) cluster analysis of groups-separated input data and (2) cluster analysis of covariate-adjusted input data.

The groups-separated analysis explored whether the cluster structure identified in the overall sample was representative of potential cluster structures in SZ-only and HC-only data examined in isolation. The prepared overall sample data that were provided as input to the above-described original cluster analysis were transformed with the variable weights that resulted from the original sparse *k*-means clustering. The weight-transformed dataset was then split by diagnostic group. The SZ-only and HC-only datasets were separately subjected to standard *k*-means clustering (using the RSKC package with the sparsity parameter *l* modified accordingly) with the number of clusters set to (1) the optimal *k* determined for the overall sample data and, if different, (2) the optimal *k* determined by reapplying the Clest, gap, and weighted gap methods separately to these SZ-only and HC-only datasets. The resulting solutions underwent bootstrapped cluster-wise stability assessment. Cluster labels for each participant were compared between the groups-separated solutions versus the original groups-combined solution to determine degree of disagreement, quantified by classification error rate and variation of information^74^.

The covariate-adjusted analysis explored whether the cluster structure in the original solution would persist after accounting for extraneous factors that may have unduly affected task performance or influenced task performance-based clustering. The raw task data underwent re-preparation to produce a covariate-adjusted version of the dataset provided as input to sparse *k*-means clustering for the original analysis. First, the power transformation parameter for each task variable was determined based on a linear regression model that included the task variable (response) and covariates (predictors)—the transformation parameter estimation then aims to normalize the residuals from the regression of the transformed response on the predictors. (This step is equivalent to the corresponding data preparation step for the original analysis, except that with no covariate adjustment in the original analysis the transformation was estimated for a null model with no predictors, in which case the residuals equal the variable centered to its mean). Each predictor in the model was the interaction effect between diagnostic group and a covariate, such that the model would estimate SZ-specific and HC-specific effects for each covariate (and thus calculate residuals centered around group-specific regression lines) alongside a common intercept. For any covariate that was measured only for SZ participants (e.g., medication dosage and illness duration) the variable was assigned a constant value of zero across all HC participants, such that the model did not exclude HC participants outright, but an HC-specific effect of the covariate could not be estimated to influence the residuals in the HC group. The models for all task variables included the same covariates, which were selected based on the results of statistical analyses of the original, unadjusted data (described in the following section). Each transformed task variable was then fitted by its linear regression model (with the same predictors as the model that had been used for transformation parameter estimation), and the residuals of each model were extracted to represent covariate-adjusted transformed task variables, which were then centered to mean and scaled by standard deviation prior to input to sparse *k*-means clustering. The number of clusters was set to (1) the optimal *k* determined for the original input dataset and, if different, (2) the optimal *k* determined by reapplying the Clest, gap, and weighted gap methods to this dataset. The remaining parameters for sparse *k*-means clustering using the RSKC package were left unaltered from the original analysis. Using similar methods as for the groups-separated analysis, the resulting solutions underwent stability assessment as well as comparison versus the original solution to quantify disagreement in cluster labels across participants. Further, we re-evaluated the statistical comparisons across clusters (described in the following section) for the measures selected as covariates (to verify that cluster-wise differences in these potential confounders had been substantively redressed by covariate-adjusted clustering); the task performance measures (to verify that cluster-wise behavioural differences had persisted despite covariate-adjusted clustering); and the primary clinical measures (to re-test our primary hypotheses regarding cluster-wise differences in motivation and functioning following covariate-adjusted clustering).

### Statistical analysis

All analyses were conducted using R version 3.3.2^72^.

#### Comparisons across diagnostic groups

Continuous demographic, clinical, and task-related measures were compared across diagnostic groups with Wilcoxon-Mann-Whitney tests using the R package “coin” version 1.2–1^75^. Categorical comparisons were conducted with the minimum likelihood-based two-sided Fisher’s exact test using the R package “exact2x2” version 1.6.5^76^.

Diagnostic group differences in Novelty Exploration Task performance were evaluated with separate multivariate analysis of covariance (MANCOVA) tests (using the R package “car”) for locomotion, visual object exploration, and tactile object exploration. Object novelty and interest scores were included as covariates due to the potential influence of individual differences in object perception on task performance, consistent with our prior analyses of Novelty Exploration Task performance^28^. Group comparison of Activity Preference Task variables was similarly performed using a multivariate analysis of variance (MANOVA) test with all four Activity Preference Task measures. Follow-up univariate analysis of (co)variance (ANCOVA or ANOVA) tests were performed for any multivariate model indicating a significant omnibus group effect (threshold at *α* = 0.05), and effect size indicators eta-squared (*η*^2^) or partial eta-squared (*η*_p_^2^) were computed using the R package “heplots” version 1.3–3. Empirical *P*-values for the effect of diagnostic group in these models were computed by 50,000-fold permutation testing of the Pillai’s trace statistic (*V*) or *F*-statistic using the Freedman-Lane procedure^77,78^.

#### Correlations with task performance

Relationships between Novelty Exploration and Activity Preference Task performance and appropriate clinical characteristics and task-related measures in the overall sample were assessed by spearman correlations (using the R package “Hmisc” version 4.3–0). Each correlation statistic was computed using all non-missing observations for the given pair of variables. The false discovery rate for each statistic amongst these multiple correlation tests was evaluated based on its *q*-value (the false discovery rate when said statistic is considered significant), based on the overall proportion of true null *P*-values estimated by bootstrapped sampling of all *P*-values in the correlation matrix (using the R package “qvalue” version 2.15.0)^79,80^. We examined the false discovery rate incurred if all correlations with *P* < 0.05 were considered significant, and identified which correlations would remain significant under a fixed control criteria of *q* < 0.05 (i.e., false discovery rate of 5%). We similarly evaluated correlations in the SZ sample between task performance versus clinical measures, object novelty and interest scores, and finger tapping performance. Partial correlations were evaluated using the R package “ppcor” version 1.1^81^.

#### Comparisons across clusters

Fisher’s exact tests evaluated whether the cluster solution predicted diagnostic group or sex (using the “exact2x2” package for any two-by-two test, as above). Bootstrapped statistical comparison tests were implemented using the R package “boot” version 1.3–20. Differences in age, the primary and secondary clinical measures, and the task measures across clusters were evaluated using one-way ANOVA tests. To compute empirical *P*-values (*P*_boot_), observed *F*-statistics were compared against null *F*-statistics generated by 50,000-fold cluster-stratified nonparametric bootstrapped resampling of the response with between-cluster mean differences nullified. Corrections for multiple comparisons were applied to the one-way omnibus tests for (1) the task measures, which were Bonferroni-corrected for 10 tests, and (2) the primary clinical measures, which were Bonferroni-corrected for four tests. Grouping the measures for correction in this manner provided conservative control of type I error rates for (1) comparisons that aimed to simply confirm that the identified behavioural phenotypes were statistically distinct—indeed, task performance differences were highly anticipated, and perhaps even obvious, because clustering was performed to differentiate subgroups using derivatives of these same task measures as input—and (2) comparisons that aimed to test our primary hypotheses with respect to clinical characteristics, without conflating these sets of corrections. No correction was applied for the remaining demographic, secondary, or exploratory analyses. Significant omnibus cluster effects (threshold at *α* = 0.05) were further evaluated post-hoc via pairwise mean differences (∆mean) and bootstrapped (bias-corrected and accelerated) 95% confidence intervals (CI), without and with Bonferroni correction (e.g., 98.3% CI for three post-hoc contrasts). Similar methods were used for any supplementary analyses of between-cluster differences in only the SZ sample.

Diagnostic group by cluster interaction effects were evaluated for age and the task and appropriate clinical measures (i.e., excluding schizophrenia-specific clinical measures, and the sample size limited BFI, DPB, BIS, and BAI), using bootstrapped two-way ANOVA tests. Null data for *F*-statistic distributions to compute *P*_*boot*_ were generated by summing cell-stratified resampled residuals from the full model and fitted values from a reduced model (including diagnosis and cluster main effects, but not their interaction)^82^. Interactions were further evaluated post-hoc via bootstrapped within-cluster SZ versus HC contrasts.

## Supplementary information

Supplement

## Data Availability

The data generated and analyzed for this study are available from the corresponding author upon reasonable request. The data are not publicly available due to research participant privacy and consent restrictions.

## Code availability

Code scripts and functions used for data processing, analysis, and visualization for this study are available from the corresponding author upon reasonable request.
