# Supplementary material for: Behavioural phenotypes of intrinsic motivation in schizophrenia determined by cluster analysis of objectively quantified real-world performance
Source: Schizophrenia (Heidelb). 2022 Oct 21;8(1):85. doi: 10.1038/s41537-022-00294-0 (PMC9587030; doi:10.1038/s41537-022-00294-0)
Supplement: Supplementary file 1 — Supplement [file 41537_2022_294_MOESM1_ESM.pdf]

Supplementary Figure S1. Cluster centers of the three-cluster sparse *k*-means solution described by behavioural task performance

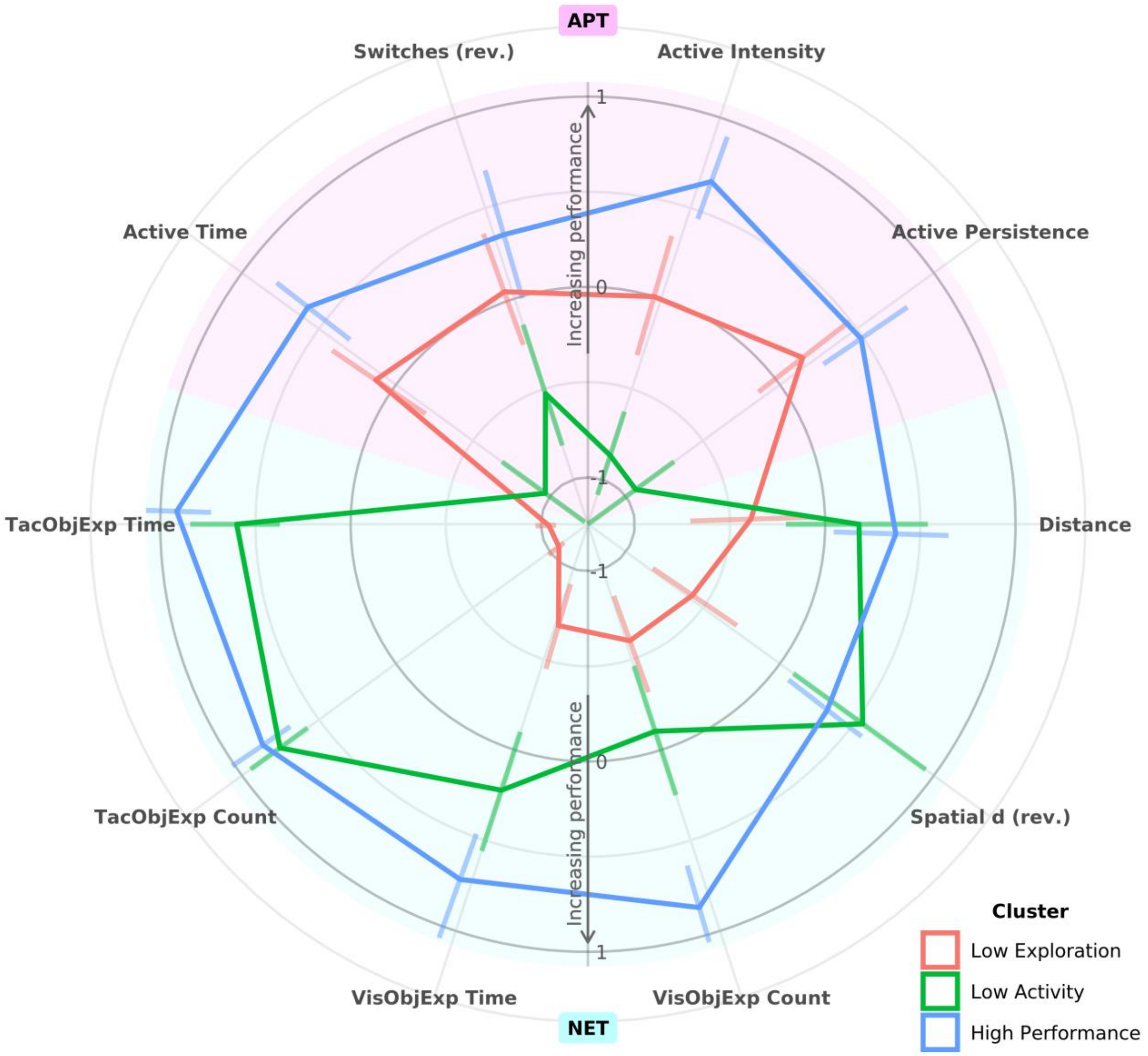

Cluster-wise means and bootstrapped (bias-corrected and accelerated) 95% confidence intervals are shown for the clustering input variables, derived from the Activity Preference Task (APT) and Novelty Exploration Task (NET). All variables are shown on a common scale such that higher values (radially outward) indicate increasing performance, with appropriate variables being reverse scaled (rev.). Based on their characteristic low NET (with medium APT) performance, low APT (with medium NET) performance, and high APT and NET performance, we respectively refer to the clusters as Low Exploration, Low Activity, and High Performance.

Task variable (sparse *k*-means weight): APT Active Time (0.27), duration on active engagement option; Switches (0.07), number of switches between active and passive engagement options, reversed; Active Intensity (0.25), average hand speed during periods of active engagement; Active Persistence (0.25), index of tendency to sustain continuous active engagement; NET Distance (0.09), total distance travelled; Spatial *d* (0.17), index of complexity of locomotion, reversed; VisObjExp Count (0.26), number of objects visually explored; VisObjExp Time (0.27), duration of visual object exploration; TacObjExp Count (0.56), number of objects physically explored; TacObjExp Time (0.55), duration of physical object exploration.

Supplementary Table S1. Overall sample (n=92) correlations (ρ) between behavioural task performance and clinical, personality, and task-related measures

	Novelty Exploration Task (NET)						Activity Preference Task (APT)			
	Locomotion		Visual Object Exploration		Tactile Object Exploration		Active Time	Switches	Active Intensity	Active Persistence
	Distance	Spatial *d*	Count	Time	Count	Time				
Age	-0.19	0.26*	-0.24*	-0.23*	-0.17	-0.08	-0.02	-0.22*	-0.05	0.01
AES	-0.10	0.16	-0.25*	-0.21*	-0.16	-0.19	-0.37**†	0.14	-0.50**†	-0.39**†
BACS Z-score	-0.01	-0.05	0.21*	0.12	0.07	0.07	0.08	-0.04	0.33**†	0.12
PSP	0.06	-0.22*	0.21*	0.10	0.09	0.11	0.21*	-0.09	0.40**†	0.28**
SFS	0.04	-0.09	0.14	0.22*	0.12	0.19	0.25*	-0.12	0.23*	0.21*
TEPS-Ant	0.04	-0.04	0.09	0.00	0.21	0.18	0.18	0.11	0.15	0.17
TEPS-Con	-0.13	0.04	0.13	0.16	0.30**	0.33**†	0.22*	-0.01	0.20	0.15
DPB	-0.02	-0.17	0.17	-0.03	-0.12	-0.11	0.11	0.03	-0.15	0.05
BIS	0.05	-0.01	-0.03	0.07	0.01	-0.06	-0.04	0.19	-0.46**†	-0.27
BAI	-0.05	0.08	-0.07	-0.05	0.03	0.02	-0.15	0.21	-0.25	-0.22
BFI-Extraversion	0.30*	-0.29*	0.23	0.25	0.27	0.12	-0.11	0.34*	0.00	-0.30*
BFI-Agreeableness	-0.19	0.12	-0.17	-0.03	0.15	0.14	0.25	-0.28*	0.19	0.32*
BFI-Conscientiousness	-0.01	-0.01	0.05	0.00	0.19	0.15	0.09	-0.27	0.34*	0.26
BFI-Neuroticism	0.10	-0.09	-0.05	-0.22	-0.08	-0.08	-0.03	0.04	-0.16	-0.01
BFI-Openness	0.09	-0.09	-0.04	0.04	0.19	0.18	0.11	0.11	0.01	-0.00
Pre-task Fatigue	-0.08	0.16	-0.00	-0.07	-0.05	-0.10	-0.01	0.09	-0.06	-0.02
NET Object Novelty	0.31**†	-0.24*	0.18	0.03	0.17	0.07				
NET Object Interest	0.30**†	-0.29**	0.29**	0.28**	0.32**†	0.19				
NET Anxiety Magnitude	0.00	-0.01	-0.17	-0.23	-0.09	-0.15				
NET Anxiety Duration	0.07	0.02	-0.24	-0.32*	-0.11	-0.20				
Post-task Fatigue							-0.07	0.03	-0.11	-0.06
APT Anxiety Level							0.03	0.04	-0.12	0.07
APT Interest Level							0.41**†	-0.22	0.25	0.37**
Finger Tapping Task							0.08	-0.00	0.23*	0.07

AES, Apathy Evaluation Scale; BACS, Brief Assessment of Cognition in Schizophrenia; PSP, Personal and Social Performance Scale; SFS, Social Functioning Scale; TEPS, Temporal Experience of Pleasure Scale (Ant, Anticipatory; Con, Consummatory); DPB, defeatist performance beliefs; BIS, Barratt Impulsiveness Scale; BAI, Beck Anxiety Inventory; BFI, Big Five Inventory.

* *P*<0.05; ** *P*<0.01; † q<0.05, false discovery rate <5% (q=0.168 if all correlations with *P*<0.05 are considered significant).

Supplementary Table S2. Schizophrenia sample (n=45) correlations (ρ) between behavioural task performance and clinical and selected task-related measures

	Novelty Exploration Task (NET)						Activity Preference Task (APT)			
	Locomotion		Visual Object Exploration		Tactile Object Exploration		Active Time	Switches	Active Intensity	Active Persistence
	Distance	Spatial *d*	Count	Time	Count	Time				
Age	-0.33*†	0.38*†A	-0.38*†A	-0.33*	-0.35*†	-0.27	-0.17	-0.25	-0.28	-0.08
Illness Duration	-0.35*†A	0.44**†A	-0.34*†	-0.42**†A	-0.36*†	-0.38*†A	-0.08	-0.19	-0.23	-0.06
CPZ Equivalents	-0.21	0.22	-0.30*	-0.06	-0.31*	-0.34*†	-0.37*†	-0.02	-0.34*†	-0.17
SAPS	-0.19	0.09	-0.03	-0.18	-0.19	-0.22	0.23	-0.24	0.14	0.19
SANS	-0.22	0.33*†	-0.26	-0.29	-0.30*	-0.27	-0.30*	-0.35*†	-0.48**†A	-0.21
SANS-DimExp	-0.11	0.14	-0.12	-0.16	-0.17	-0.17	-0.21	-0.26	-0.38*†	-0.12
SANS-Amot	-0.31*	0.41**†	-0.35*†	-0.29	-0.37*†	-0.31*	-0.31*	-0.35*†	-0.47**†A	-0.24
CDSS	-0.09	0.04	-0.18	0.03	-0.11	-0.09	-0.10	0.01	-0.00	0.02
SAS	0.03	0.04	-0.19	-0.09	-0.24	-0.25	-0.07	0.15	-0.37*†A	-0.16
AES	-0.20	0.33*	-0.30*	-0.33*	-0.31*	-0.26	-0.37*†	-0.25	-0.34*†	-0.18
BACS Z-score	-0.10	-0.06	0.16	0.10	0.04	0.07	-0.20	0.32*	0.12	-0.24
PSP	0.24	-0.42**†	0.31*	0.25	0.36*†	0.34*†	0.26	0.26	0.37*†	0.27
SFS	0.22	-0.23	0.35*†	0.31*	0.26	0.24	0.26	0.23	0.23	0.04
TEPS-Ant	-0.08	-0.04	0.00	-0.08	0.17	0.15	0.21	0.09	0.10	0.19
TEPS-Con	-0.15	-0.14	0.25	0.19	0.41**†A	0.43**†A	0.27	0.30	0.21	0.09
NET Object Novelty	0.11	-0.01	0.01	0.01	0.07	0.06				
NET Object Interest	0.20	-0.31*	0.40**†A	0.35*†	0.42**†A	0.34*†				
Finger Tapping Task							-0.07	0.15	0.12	-0.04

CPZ, chlorpromazine dose; SAPS, Scales for the Assessment of Positive Symptoms; SANS, Scales for the Assessment of Negative Symptoms (DimExp, Diminished Expression subdomain; Amot, Amotivation subdomain); CDSS, Calgary Depression Scale for Schizophrenia; SAS, Simpson Angus Rating Scale; AES, Apathy Evaluation Scale; BACS, Brief Assessment of Cognition in Schizophrenia; PSP, Personal and Social Performance Scale; SFS, Social Functioning Scale; TEPS, Temporal Experience of Pleasure Scale (Ant, Anticipatory; Con, Consummatory).

* $P<0.05$; ** $P<0.01$; † q<0.05, false discovery rate <5% (q=0.062 if all correlations with $P<0.05$ are considered significant); A significant ($P<0.05$) zero-order ρ that remains significant ($P<0.05$) in partial correlation controlled for AES.

Supplementary Table S3. Behavioural task performance and clinical characteristics across clusters

	Low Exploration (LE) Cluster (n=39) mean ($CI_{95\%}$)	Low Activity (LA) Cluster (n=23) mean ($CI_{95\%}$)	High Performance (HP) Cluster (n=30) mean ($CI_{95\%}$)	Fisher's Exact Test / One-way ANOVA Cluster Effect	Post-hoc Contrasts, contrast=Δmean ($CI_{95\%}$)
APT Active Time[1]	563.74 (470.77, 644.75)	249.57 (174.26, 332.12)	714.27 (632.12, 775.27)	F(2,88)=25.96, P<0.001†, P_{boot}<0.001†	LE–LA=314.17 (192.25, 426.79)*†<br>LE–HP=-150.53 (-263.32, -39.66)*†<br>LA–HP=-464.70 (-562.34, -351.46)*†
APT Switches[1]	9.16 (6.77, 12.74)	14.70 (10.87, 19.61)	6.93 (4.70, 11.40)	F(2,88)=4.58, P=0.013, P_{boot}=0.015	LE–LA=-5.54 (-10.73, -0.37)*<br>LE–HP=2.22 (-2.13, 6.25)<br>LA–HP=7.76 (2.37, 12.90)*†
APT Active Intensity[1]	1.26 (1.06, 1.47)	0.73 (0.59, 0.88)	1.68 (1.55, 1.83)	F(2,88)=22.68, P<0.001†, P_{boot}<0.001†	LE–LA=0.54 (0.29, 0.78)*†<br>LE–HP=-0.41 (-0.66, -0.17)*†<br>LA–HP=-0.95 (-1.15, -0.76)*†
APT Active Persistence[1]	4.15 (3.70, 4.73)	2.46 (2.12, 2.77)	4.61 (4.21, 5.24)	F(2,88)=16.75, P<0.001†, P_{boot}<0.001†	LE–LA=1.69 (1.13, 2.36)*†<br>LE–HP=-0.46 (-1.18, 0.24)<br>LA–HP=-2.15 (-2.83, -1.63)*†
NET Distance[1]	98.53 (75.87, 129.06)	143.06 (108.08, 194.96)	158.14 (128.80, 196.24)	F(2,89)=3.83, P=0.025, P_{boot}=0.031	LE–LA=-44.54 (-97.16, 2.35)<br>LE–HP=-59.62 (-102.27, -17.70)*†<br>LA–HP=-15.08 (-66.72, 40.60)
NET Spatial *d*[1]	1.40 (1.36, 1.44)	1.29 (1.26, 1.36)	1.29 (1.27, 1.32)	F(2,89)=11.90, P<0.001†, P_{boot}<0.001†	LE–LA=0.11 (0.04, 0.17)*†<br>LE–HP=0.11 (0.07, 0.16)*†<br>LA–HP=0.00 (-0.04, 0.07)
NET VisObjExp Count[1]	5.10 (4.28, 5.85)	6.50 (5.36, 7.27)	8.70 (8.20, 9.03)	F(2,88)=24.47, P<0.001†, P_{boot}<0.001†	LE–LA=-1.40 (-2.59, -0.14)*<br>LE–HP=-3.60 (-4.49, -2.71)*†<br>LA–HP=-2.20 (-3.31, -1.25)*†
NET VisObjExp Time[1]	57.63 (44.68, 78.37)	132.65 (103.73, 172.80)	189.21 (159.56, 243.78)	F(2,88)=21.82, P<0.001†, P_{boot}<0.001†	LE–LA=-75.01 (-114.95, -39.80)*†<br>LE–HP=-131.57 (-184.49, -96.94)*†<br>LA–HP=-56.56 (-114.96, -10.13)*†
NET TacObjExp Count[1]	0.03 (0.00, 0.08)	1.95 (1.55, 2.45)	2.23 (1.77, 2.87)	F(2,88)=46.36, P<0.001†, P_{boot}<0.001†	LE–LA=-1.93 (-2.50, -1.57)*†<br>LE–HP=-2.21 (-2.87, -1.77)*†<br>LA–HP=-0.28 (-1.02, 0.37)
NET TacObjExp Time[1]	0.11 (0.05, 0.25)	26.39 (13.07, 73.95)	33.27 (24.21, 44.34)	F(2,88)=10.58, P<0.001†, P_{boot}=0.012	LE–LA=-26.28 (-73.83, -12.96)*†<br>LE–HP=-33.16 (-44.22, -24.11)*†<br>LA–HP=-6.88 (-23.59, 32.81)
Group,[2] n_{SZ} : n_{HC}	20 (51%) : 19 (49%)	15 (65%) : 8 (35%)	10 (33%) : 20 (67%)	P=0.067	
Sex,[2] n_{female} : n_{male}	15 (38%) : 24 (62%)	8 (35%) : 15 (65%)	13 (43%) : 17 (57%)	P=0.827	
Age,[2] years	36.85 (33.49, 40.23)	32.13 (27.61, 37.61)	34.20 (31.07, 37.57)	F(2,89)=1.45, P=0.241, P_{boot}=0.246	
AES[3]	29.72 (27.44, 32.44)	31.83 (29.17, 34.78)	25.33 (23.83, 27.43)	F(2,89)=6.31, P=0.003†, P_{boot}=0.003†	LE–LA=-2.11 (-5.77, 1.69)<br>LE–HP=4.38 (1.43, 7.55)*†<br>LA–HP=6.49 (3.21, 9.82)*†
BACS Z-score[3]	-0.46 (-1.00, 0.00)	-0.53 (-1.04, -0.03)	0.05 (-0.46, 0.52)	F(2,87)=1.41, P=0.250, P_{boot}=0.232	
PSP[3]	65.33 (59.13, 71.33)	64.35 (57.17, 70.52)	71.97 (68.30, 75.47)	F(2,89)=1.86, P=0.161, P_{boot}=0.141	
SFS[3]	116.18 (112.93, 118.85)	114.21 (110.29, 117.47)	119.80 (117.15, 121.89)	F(2,89)=3.03, P=0.054, P_{boot}=0.051	
TEPS-Ant[4]	44.11 (41.89, 46.20)	43.05 (39.63, 46.53)	46.89 (44.39, 49.07)	F(2,79)=2.11, P=0.128, P_{boot}=0.145	
TEPS-Con[4]	35.97 (33.69, 37.83)	37.58 (35.63, 39.47)	40.61 (38.25, 42.25)	F(2,79)=5.35, P=0.007, P_{boot}=0.005	LE–LA=-1.61 (-4.51, 1.06)<br>LE–HP=-4.64 (-7.46, -1.75)*†<br>LA–HP=-3.03 (-5.58, -0.07)*
DPB[4]	41.11 (32.67, 49.28)	39.23 (31.85, 48.00)	34.16 (28.79, 39.74)	F(2,47)=0.97, P=0.387, P_{boot}=0.384	
BIS[4]	58.17 (53.28, 63.28)	64.08 (60.77, 68.08)	54.10 (50.75, 59.40)	F(2,48)=4.21, P=0.021, P_{boot}=0.020	LE–LA=-5.91 (-12.04, 0.33)<br>LE–HP=4.07 (-2.81, 10.22)<br>LA–HP=9.98 (3.85, 15.07)*†
BAI[4]	5.33 (3.28, 8.22)	12.85 (7.54, 20.31)	3.65 (2.35, 5.50)	F(2,48)=6.96, P=0.002, P_{boot}=0.031	LE–LA=-7.51 (-15.04, -1.62)*†<br>LE–HP=1.68 (-1.03, 4.82)<br>LA–HP=9.20 (3.67, 16.76)*†
BFI-Extraversion[4]	23.56 (20.79, 25.94)	24.62 (20.85, 28.15)	25.80 (22.40, 28.70)	F(2,48)=0.52, P=0.595, P_{boot}=0.589	
BFI-Agreeableness[4]	36.89 (34.06, 39.22)	35.85 (33.69, 38.15)	39.00 (36.85, 40.80)	F(2,48)=1.77, P=0.181, P_{boot}=0.171	
BFI-Conscientiousness[4]	33.50 (31.33, 35.94)	31.31 (29.31, 34.62)	37.25 (34.00, 39.60)	F(2,48)=4.82, P=0.012, P_{boot}=0.014	LE–LA=2.19 (-1.44, 5.38)<br>LE–HP=-3.75 (-7.04, 0.14)<br>LA–HP=-5.94 (-9.21, -1.67)*†
BFI-Neuroticism[4]	20.56 (17.17, 23.67)	23.00 (20.08, 25.38)	17.95 (15.00, 20.60)	F(2,48)=2.46, P=0.096, P_{boot}=0.084	
BFI-Openness[4]	34.83 (30.61, 38.06)	36.62 (33.54, 40.31)	38.70 (36.20, 41.35)	F(2,48)=1.46, P=0.242, P_{boot}=0.238	

[1] Behavioural task measures: APT, Activity Preference Task; Active Time, duration (s) on active engagement option; Switches, number of switches between active and passive engagement options; Active Intensity, average hand speed (m/s) during periods of active engagement; Active Persistence, index of tendency to sustain continuous active engagement. NET, Novelty Exploration Task; Distance, total distance travelled (m); Spatial *d*, index of complexity of locomotion – lower values indicate more linear, less circumscribed movement; VisObjExp, visual object exploration (Count, number of objects explored; Time, duration (s) of exploration); TacObjExp, tactile object exploration (Count, number of objects explored; Time, duration (s) of exploration). (One-way omnibus tests Bonferroni-corrected for 10 tests.)

[2] Case-control matching measures. SZ, schizophrenia group; HC, healthy control group.

[3] Primary clinical measures: AES, Apathy Evaluation Scale; BACS, Brief Assessment of Cognition in Schizophrenia; PSP, Personal and Social Performance Scale; SFS, Social Functioning Scale. (One-way omnibus tests Bonferroni-corrected for four tests.)

[4] Secondary/Exploratory measures (administered to subsamples): TEPS, Temporal Experience of Pleasure Scale (Ant, Anticipatory; Con, Consummatory); DPB, defeatist performance beliefs; BIS, Barratt Impulsiveness Scale; BAI, Beck Anxiety Inventory; BFI, Big Five Inventory.

P_{boot}, *P*-value determined by omnibus F-statistic bootstrapping († P_{boot}<0.05 following Bonferroni correction); Δmean, mean difference; CI, bootstrapped (bias-corrected and accelerated) confidence interval; * 95% bootstrapped CI indicating significant (non-zero) mean difference for post-hoc contrast, evaluated only if omnibus P_{boot}<0.05 († 95% CI, with Bonferroni correction for three post-hoc contrasts, indicating significant mean difference).

Supplementary Table S4. Confusion matrices comparing alternative cluster analytic classifications of participants separated by diagnostic group

	Groups-combined sparse 3-means clustering[1]								
	SZ Group				HC Group				
	Cluster label (n)	LE (20)	LA (15)	HP (10)	Error / Variation	Cluster label (n)	LE (19)	LA (8)	HP (20)	Error / Variation
Groups-separated 2-means clustering	**LE**[2] (19)	19	0	0	CER=0.20 VI=0.56	**LE**[3] (21)	19	1	1	CER=0.18 VI=0.64
	**non-LE**[2] (26)	1	15	10		**non-LE**[3] (26)	0	7	19	
Groups-separated 3-means clustering	**LP**[4] (8)	6	2	0	CER=0.29 VI=1.02	**LE**[5] (19)	19	0	0	CER=0.02 VI=0.15
	**LE**[4] (13)	13	0	0		**LA**[5] (9)	0	8	1	
	**non-LE**[4] (24)	1	13	10		**HP**[5] (19)	0	0	19	

[1] Cluster labels in the original three-cluster (k=3) solution comprising Low Exploration (LE), Low Activity (LA), and High Performance (HP) clusters are tabulated separately for Schizophrenia (SZ) and Healthy Control (HC) participants to allow comparison versus cluster solutions produced separately for each diagnostic group. Cluster label disagreement between solutions are quantified by classification error rate (CER) and variation of information (VI), with higher values indicating greater disagreement.

[2] For SZ, clustering with k=2 was deemed optimal by gap and weighted gap statistics, but not by Clest (with P=0.074 for k=2 against the null hypothesis k=1). Compared to the original solution, the resulting clusters roughly differentiated LE and non-LE (i.e., mainly LA and HP) participants. Bootstrapped assessment indicated both clusters were highly stable (mean Jaccard similarities of 0.979 and 0.983 respectively).

[3] For HC, clustering with k=2 was deemed optimal by gap and weighted gap statistics, and acceptable (but not optimal) by Clest. Compared to the original solution, the resulting clusters roughly differentiated LE and non-LE (i.e., mainly LA and HP) participants. Bootstrapped assessment indicated both clusters were highly stable (mean Jaccard similarities of 0.994 and 0.995 respectively).

[4] For SZ, clustering with k=3 was not recommended by Clest, gap, or weighted gap methods, and was only evaluated because k=3 had been deemed optimal for the original groups-combined solution. Compared to the original solution, the resulting clusters roughly differentiated Low Performance (LP; i.e., only LE and LA participants), LE, and non-LE (i.e., mainly LA and HP) participants. Bootstrapped assessment indicated LP was unstable or suspect, but LE and non-LE were acceptably stable (mean Jaccard similarities of 0.582, 0.818, and 0.792 respectively).

[5] For HC, clustering with k=3 was deemed optimal by Clest, but not by gap and weighted gap statistics. Compared to the original solution, the resulting clusters almost identically differentiated LE, LA, and HP participants. Bootstrapped assessment indicated the clusters were highly stable (mean Jaccard similarities of 0.974, 0.907, and 0.954 respectively).

Supplementary Table S5. Confusion matrices comparing alternative covariate-adjusted cluster analytic classifications of participants

		Original sparse 3-means clustering[1]			
	Cluster label (n)	**LE** (39)	**LA** (23)	**HP** (30)	**Error / Variation**
**Covariate-adjusted sparse 3-means clustering[2]**	**LE** (38)	36	1	1	CER=0.13 VI=0.78
	**LA** (21)	2	18	1	
	**HP** (33)	1	4	28	
**Covariate-adjusted sparse 4-means clustering[3]**	**LEHA** (15)	14	0	1	CER=0.18 VI=0.97
	**LE** (24)	23	1	0	
	**LA** (20)	1	18	1	
	**HP** (33)	1	4	28	

[1] Cluster labels in the original three-cluster (*k*=3) solution comprising Low Exploration (LE), Low Activity (LA), and High Performance (HP) clusters are compared versus cluster solutions produced with input data consisting task variables adjusted for participant age, illness duration, and chlorpromazine dose equivalents. Cluster label disagreement between solutions are quantified by classification error rate (CER) and variation of information (VI), with higher values indicating greater disagreement.

[2] Clustering with *k*=3 was deemed optimal by gap and weighted gap statistics, and acceptable (but not optimal) by Clest. The resulting clusters roughly aligned with the original LE, LA, and HP clusters, and are named accordingly. Bootstrapped assessment indicated LA was acceptably stable, and LE and HP were highly stable (mean Jaccard similarities of 0.760, 0.886, and 0.894 respectively).

[3] Clustering with *k*=4 was deemed optimal by Clest, but not by gap and weighted gap statistics. The resulting clusters roughly aligned with two subdivisions of the original LE cluster – behaviourally distinguishable as Low Exploration (LE) versus Low Exploration/High Activity (LEHA) – and the original LA and HP clusters, and are named accordingly. Bootstrapped assessment indicated LEHA and LE were acceptably stable, and LA and HP were highly stable (mean Jaccard similarities of 0.768, 0.779, 0.883, 0.940 respectively).
